# Discovery of an algicidal compound from *Brevibacterium* sp. BS01 and its effect on a harmful algal bloom-causing species, *Alexandrium tamarense*

**DOI:** 10.3389/fmicb.2015.01235

**Published:** 2015-11-05

**Authors:** Xinli An, Bangzhou Zhang, Huajun Zhang, Yi Li, Wei Zheng, Zhiming Yu, Lijun Fu, Tianling Zheng

**Affiliations:** ^1^State Key Laboratory of Marine Environmental Science and Key Laboratory of Ministry of Education for Coast and Wetland Ecosystems, School of Life Sciences, Xiamen UniversityXiamen, China; ^2^Institute of Urban Environment, Chinese Academy of SciencesXiamen, China; ^3^Key Laboratory of Marine Ecology and Environmental Science, Institute of Oceanology, Chinese Academy of SciencesQingdao, China; ^4^Department of Environment and Life Science, Putian UniversityPutian, China

**Keywords:** algicidal compound, *Brevibacterium* sp. BS01, *Alexandrium tamarense*, algicidal effect, harmful algal bloom control

## Abstract

Blooms of the dinoflagellate *Alexandrium tamarense* have become worldwide phenomena and have detrimental impacts on aquatic ecosystems and human health. In this study, a culture supernatant of the marine actinomycete BS01 exerted a strong algicidal effect on *A. tamarense* (ATGD98-006). The target algicide from BS01 was separated by adsorption chromatography and identified by MALDI-TOF-MS and NMR analysis. The results suggested that the purified algicidal component corresponded to a hydrophobic compound (2-isobutoxyphenyl)amine (C_10_H_15_NO) with a molecular weight of 165 Da, which exhibited a significant algicidal effect (64.5%) on *A. tamarense*. After incubation in 5 μg/mL of (2-isobutoxyphenyl)amine for 24 h, the algae lost mobility and sank to the bottom of the flasks, and 56.5% of the algae cells lost vitality at a concentration of 20 μg/mL (*p* < 0.01) despite having intact cell profiles. Morphological analysis revealed that the cell structure of *A. tamarense* was altered by (2-isobutoxyphenyl)amine resulting in cytoplasm degradation and the loss of organelle integrity. The images following propidium iodide staining suggested that the algal nucleus was also severely damaged and eventually degraded due to exposure to the algicidal compound. All of the results indicate that (2-isobutoxyphenyl)amine from the actinomycete might be a candidate for the control of bloom-forming *A. tamarense*.

## Introduction

Harmful algal blooms (HABs) occur around the world, causing significant, direct economic losses to seafood-based industries (Anderson, 1997; Geohab, 2001), and the consumption of seafood contaminated by the toxins produced by these algae also pose potential threats to human health (Geohab, 2001). *Alexandrium tamarense* is one of the most notorious toxic HAB-causing species; it causes paralytic shellfish poisoning (PSP) in both shellfish and fish (Li et al., 2014). Currently, PSP is one of the most widely distributed and detrimental HAB toxins, and it is having enormous impacts on marine fisheries and human health (Wang et al., 2012; Li et al., 2014). Therefore, the mitigation and control of *A. tamarense* through various strategies has become a critical research topic.

Several measures have been taken to control HABs and relieve the associated damage, including the application of chemicals as well as physical methods, but these strategies are expensive or have upset the balance of aquatic ecosystems by killing beneficial plankton and fishes (Lee et al., 2008, 2013; Li and Pan, 2013). In recent years, the use of microbial agents, especially naturally occurring algicidal bacteria, to mitigate HABs has attracted global attention (Kim et al., 2007; Zheng et al., 2013). Algicidal bacteria potentially play an important role in regulating the growth, metabolism, and toxin production of harmful algae, and the inhibitory effects of algicidal bacteria have mostly been investigated to find a biological control for harmful algal blooms (Paul and Pohnert, 2011). It has been reported that most of the algicidal bacteria isolated from natural environments appear to directly or indirectly attack their target algal species (Lee et al., 2000; Bai et al., 2011; Cho, 2012). The main algicidal compounds reported in the literature include pigments (Dakhama et al., 1993; Jeong et al., 2005; Nakashima et al., 2006), peptides (Lee et al., 2000), proteins (Wang et al., 2012), amino acids (Yamamoto et al., 1998; Yoshikawa et al., 2000), antibiotics (Kawano et al., 1997; Imamura et al., 2000), alkaloids (Kodani et al., 2002; Jeong et al., 2003), hydroxylamines (Berger et al., 1979), toxins (Banin et al., 2001), and biosurfactants (Seunghak et al., 2003; Wang et al., 2005) (see Table 1). Lee et al. (2000) reported that an extracellular protease from strain A28 of the marine bacterium *Pseudoalt eromonas* was algicidal toward *Skeletonema costatum*, and algicidal compounds with lower molecular weight, such as the rhamnolipid biosurfactants from *Pseudomonas aeruginosa* or the prodigiosin pigment from the bacterium *Hahella chejuensis*, have also been identified (Wang et al., 2005; Kwon et al., 2010). Dakhama et al. (1993) found that two pigments secreted by *Pseudomonas aeruginosa*, 1-hydroxyphenazine and oxychlororaphine, strongly inhibited the growth of green microalgae and cyanobacteria. Given the considerable recent attention to the field of harmful algae, other active compounds have been screened and subsequently identified; the algicidal DHQ25, which is affiliated with the γ*-proteobacteria* subclass, produced a P7 protein that kills the toxic dinoflagellate *A. tamarense* (Wang et al., 2012). However, with few exceptions, the identities of the compounds or enzymes responsible for the algicidal effects remain unknown, and most studies continue to focus on the isolation, identification or characterization of algicidal bacteria.

**Table 1:** **The algicidal compounds reported in the literatures before 2010**.

**Category**	**Microbes**	**Target algae**	**Date**
Virus (Kay D. Bidle)	*Emiliania huxleyi Virus* 1	*Emiliania huxleyi*	2007
Prodigiosin (Takuji Nakashima; Haeyoung Jeong)	*Hahella* sp. MS-02-063	*Heterosigma akashiwo (NIES-6); Gymnodinium impudicum; Alexandrium tamarense*	2006
		*Cochlodinium polykrikoides*	2005
Rhamnolipid (Xiulin Wang)	*Pseudomonas aeruginosa*	*Heterosigma akashiwo*	2004
Bacillamide (Seong-Yun Jeong)	*Bacillus* sp. SY-1	*Cochlodinium polykrikoides*	2003
Sophorolipid (Baek Seunghak)	*Candida bombicola*	*Scripsicllu trochoidea; Prorocentrum minimum; Cochlodinium polykrikoides; Heterosigma akashishiwo;*	2003
Harmane (Shinya Kodani)	*Pseudomonas* sp. K44-1	*Anabaena cylindrica* NIES-19	2002
Toxin P (Ehud Banin)	*Vibrio shiloi* AK1	*Oculina patagonica*	2001
L-CNAla (Kazuhiro Yoshikawa)	*Vibrio* sp. C-979	*Oscillatoria amphibia* NIES 2361	2000
Serine protease (Sun-og Lee)	*Pseudoalteromonas* sp. A28	*Skeletonema costatum*	2000
Argimicin A (Nobutaka)	*Sphingomonas* sp.	Cyanobacteria	2000
L-lysine (Yoko Yamamoto)	*Streptomyces phaeofacien*	*Microcystis aeruginosa*	1998
Tiotropocin (Kawano Y)	*Caulobacter* sp. PK654	*Skeletonema costatum; Heterosigma akashiwo*	1997
1-hydroxyphenazine; oxychlororaphine (A. Dakhama)	*Pseudomonas aeruginosa*	Green microalgae, cyanobacteria	1993
hydroxylamine-N (Paul S. Berger)	*Arthrobacter* sp.	*Chlorella vulgaris*	1979

It is well known that actinomycetes produce active compounds, such as antibiotics, enzymes, organic acids and so on (Yamamoto et al., 1998; Hee-Jin et al., 2005; Zhang et al., 2014). Due to their successful use as antibiotics in medical science, actinomycetes have been neglected as one of the most crucial potential biological algicides for the regulation and control of red tides and the maintenance of the balance of marine ecosystems. To our knowledge, there have been no reports of algicidal compounds identified from actinomycetes that are effective against dinoflagellates, especially *A. tamarense*. On the other hand, although the effects of algicidal extracts on the chloroplast and nuclear function of algal cells have been reported, the toxicity of pure algicidal compounds to algal cells has not been studied in *A. tamarense* (Lee et al., 2000; Wang et al., 2005; Paul and Pohnert, 2011; Li et al., 2014).

Our previous research described the isolation, identification and characterization of the algicidal actinomycete *Brevibacterium* sp. BS01, which exhibited strong algicidal activity against *A. tamarense* (Bai et al., 2011). In this study, we attempted to isolate and purify the algicidal compounds from *Brevibacterium* sp. BS01 to further uncover its algicidal mechanism and reveal some of the characteristics that may be useful for scientists working on different aspects of HABs. The objectives of our study were (1) to identify and investigate the production of algicidal compounds in BS01 and (2) to monitor the algicidal activity and process of death in *A. tamarense*.

## Materials and methods

### Culture of algae and bacteria

The target alga, *A. tamarense* ATGD98-006, was donated by the Algal Culture Collection of the Institute of Hydrobiology at Jinan University in Guangzhou, China. It was cultivated under a 12:12 h light-dark cycle with 50 μmol photons m^−2^·s^−1^ at 20 ± 1°C in a sterilized f/2 medium prepared with natural seawater (Guillard, 1975). Algal cultures in an exponential growth phase were divided into aliquots for batch experiments.

The bacterial strain BS01 (GenBank No. GQ274005), which is affiliated with the *Brevibacterium* genus of the Actinomycetales, was isolated from Xiamen Bay in China, and it exhibited a strong algicidal effect on the toxic dinoflagellate, *A. tamarense* (Bai et al., 2011). The bacterial strain was inoculated at 25°C (180 rpm for 24~48 h) in Zobell 2216E agar (Haibo, Qingdao, China), which was prepared by adding 1% agar (Difco) to Zobell 2216E medium (5 g peptone, 1 g yeast extract, and 1 g FePO_4_ dissolved in natural seawater with a pH of 7.6–7.8). Cells were removed by centrifugation at 10,000 × g for 10 min, and the supernatant was collected and stored at 4°C until use.

### Measurement of algicidal activity

The algicidal activities of the bacterial supernatant and the algicidal fragments were investigated by inoculating 99 mL of representative, exponentially growing *A. tamarense* culture with 1 mL of the bacterial supernatant (1%, v/v) or different concentrations of algicidal fragments. Control 1-mL aliquots of 2216E medium were diluted with 99 mL of the same algal culture. Flasks were cultured under the conditions described above, and the determinations were performed over regular time intervals. The total cell numbers were measured under a light microscope (Olympus BX50) after fixing the algal cells with Lugol's solution. Algal cell mortality, representing algicidal activity, was calculated using the following equation: algal cell mortality (%) = [(N_0_-N_1_)/N_0_] × 100%, where N_0_ represents the number of living algal cells in the control groups, and N_1_ refers to the number of living algal cells in the experimental groups (Bai et al., 2011).

### Size fractionation

Size fractionation experiments were performed with a filtrate of the BS01 supernatant. A total volume of 10 mL of the filtrates was fractionated using a dialysis bag with a molecular weight cutoff of 500 (MWCO 500, Lulong Biotech, China) in sterilized seawater for 48 h, according to the manufacturer's instructions. The algicidal activity of the filtrate was monitored as described above.

### Isolation and purification of the algicidal compound

The collected bacterial supernatant (1.2 L) was extracted five times at room temperature with equal volumes of ethyl acetate, and the extracts (organic phase) were incorporated and concentrated using a rotary evaporator under a vacuum. The salt and protein in the extract were precipitated three times using ethanol (100 mL) and removed by centrifugation; the resulting ethanol supernatant was evaporated and re-dissolved in 1 mL of methanol. The methanol solution (Fraction A) was then loaded on a silica gel column (granularity: 200–300 mesh, pH 6–7, 15 × 180 mm, 20 × 200 mm; BOMEX, Beijing, China), which was subsequently chromatographed using 120 mL of petroleum ether/ethyl acetate (4/0.5, 4/1.5, 4/2.5, and 4/3.5 v/v, respectively) and, finally, 200 mL of ethyl acetate. Six fractions (A_1_~A_6_) were achieved according to the thin-layer chromatograph analysis (TLC, GF254, pH 6.2–6.8; Qingdao, China), and the separated portions (fractions A_2_~A_6_) with the potential for algicidal activity (based on algal cell mortality) were repeated for incorporation and concentration. The concentrated fraction (fraction B) was subject to LH-20 gel chromatography (Sephadex™ 30 × 500 mm, Healthcare Bio-Science AB, Sweden) for further separation by elution with methanol. Of the five fractions (B_1_~B_5_) from the second separation, fraction B_2_ exhibited an obvious algicidal effect, and its analysis by TLC on silica gel produced two visualized bands (Supplementary Figure 1). The separated bands were scraped and eluted with methanol, and after concentration under a vacuum, algicidal bioassays of all of the fractions (fraction C_1_ and C_2_) were performed in the 96-well tissue culture plate according to the method described by Charles et al. (2005) (Nunc, Thermo Science, United States). The resulting portion C_2_ with strong algicidal activity was kept at −20°C for further analysis.

### MS and NMR analysis

The *m/z* ratio of the active compound (fraction C_2_) was measured by matrix-assisted laser desorption/ionization time-of-flight mass spectrometry (MALDI-TOF-MS). In brief, 0.8 μL of the purified active fraction was dipped on the ground board, and carbon nanotubes (CNT) were used as the assistant matrix. The mass spectra were then achieved with a Bruker REFLEX III mass spectrometer (Bruker, Karlsruhe, Germany). For the nuclear magnetic resonance (NMR) analysis, the purified algicidal compound was dried and dissolved in CDCl_3_, and the NMR spectra (1D proton and carbon, 2D ^1^H-^1^H and ^1^H-^13^C) were recorded by a DRX500 (BrukerBiospin Co., Karlsruhe, Germany) at 25°C. Trimethylsilyl (TMS) was used as the internal standard.

### High performance liquid chromatography (HPLC) analysis

The HPLC experiments were carried out by connecting the C18 column to an HPLC plunger pump (HITACHI L-2000, Japan). Elution was conducted by using a linear volume gradient of the mixture of methanol and H_2_O (eluting from 40%:60% to 0%:100% for 10 min and then keep the ratio of 40%:60% for 10min) with the flowing speed 1 mL/min. The standard sample (described as the below) and the culture of BS01 are monitored by measuring the ultraviolet absorption (288 nm) through a HITACHI Diode Array Detector L-2455 (Japan).

### Algicidal effect of (2-isobutoxyphenyl)amine on *A. tamarense*

Authentic (2-isobutoxyphenyl)amine was purchased from J&K Scientific Ltd. (Beijing, China) to analyze its algicidal effect against *A. tamarense*. The standard compound was dissolved in 1 mL of dimethyl sulfoxide (DMSO) to obtain the initial concentration (100 mg/mL), and the final controlled concentrations of (2-isobutoxyphenyl)amine in algal culture were 5, 10, 15, and 20 μg/mL. DMSO was used as a control to detect whether it had any algicidal effect on *A. tamarense*, and 1 mL of Zobell 2216E was added to the algal cultures as a negative control. The bacterial supernatant (cultivated for 48 h) was added to the *A. tamarense* cultures to a final concentration of 1% (v/v) and used as a positive control. Each treatment was incubated in triplicate for 24 h at 20 ± 1°C, and the *A. tamarense* cells were counted at 4, 8, 12, and 24 h.

### Cell membrane integrity and nuclear structure degradation

To observe the morphological changes in the algal cells, *A. tamarense* cultures were treated with (2-isobutoxyphenyl)amine at a final concentration of 20 μg/mL, and 1% (v/v) of the bacterial supernatant was added into the algal culture as a positive control. After 24 h of incubation, 50 μL of the algal cultures fixed by Lugol's solution were dipped on the ground slide, and the algal cells were monitored and photographed under a light microscope (10 × 100).

Propidium iodide (PI) was used for the visualization of the nuclei in the dead cells under a confocal laser scanning microscope (CLSM, Carl Zeiss AG, Germany). After treatment with (2-isobutoxyphenyl)amine (20 μg/mL), 10 mL of the algal cells were collected at 3000 × g for 5 min followed by washing with 1 mL of PBS (50 mM, pH 7.4). The algal pellets were suspended in 1 mL of PBS containing 1 μL PI (Beyotime, China) and incubated in the dark for 20 min. Algal cells were collected by centrifugation at 3000 × g for 5 min and then resuspended in 1 mL of PBS. A CLSM (Zeiss LSM 780) was used to image the algal cells through a × 401.2 N.A. water immersion objective. PI fluorescence was examined (Ex 535 nm and Em 615 nm), and representative pictures were taken.

### Statistical analysis

All data were presented as the means ± standard error of the mean (SEM). One-Way ANOVA followed by Tukey's test was used to analyze the algicidal effects of the different concentrations of (2-isobutoxyphenyl)amine on *A. tamarense* over time. Statistical significance was attained at *P* < 0.05 unless otherwise noted, and all of the analyses were performed using SPSS (19.0) software.

The SciFinder Web platform (http://scifinder.cas.org) was used to search for the molecular structure of the chemical compound, which was drawn with the ChemDraw drawing editor based on the NMR spectrum analysis.

## Results

### Identification of (2-isobutoxyphenyl)amine

**Figure 1:**
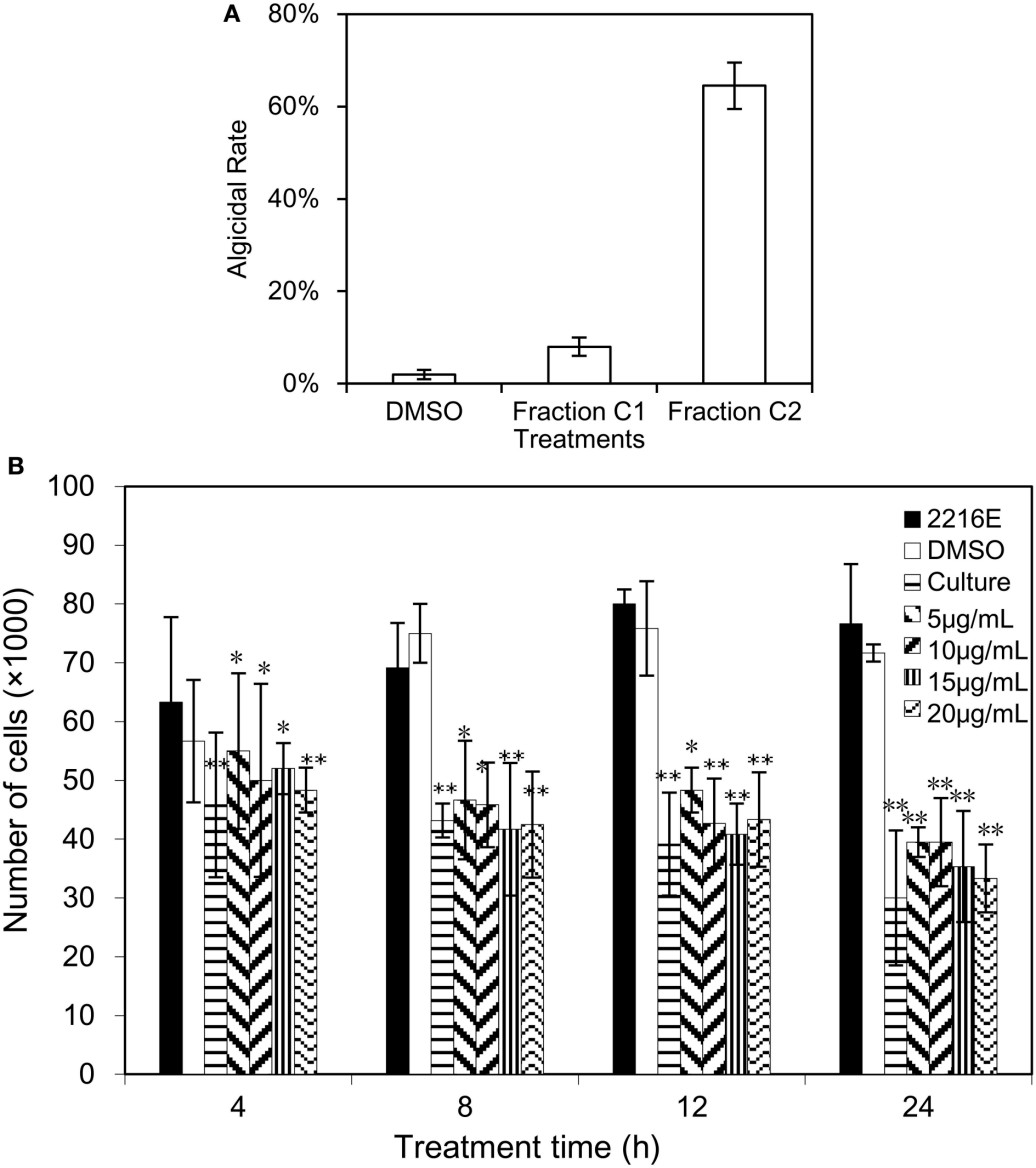
**Algicidal assay of fraction C_2_ and (2-isobutoxyphenyl)amine**. **(A)** The algicidal effect of fraction C_2_ on *A. tamarense*; **(B)** the algicidal activity of bacterial culture and (2-isobutoxyphenyl)amine on *A. tamarense*. All error bars correspond to the standard deviation. ^*^Represents a statistically significant difference of *p* < 0.05 compared to the control, and ^**^Represents a statistically significant difference of *p* < 0.01.

Due to the density difference between the supernatant and seawater, the supernatant will be diluted, resulting in the low concentration of the algicidal compound in the dialysis bag. Given that the dilution rate of the supernatant in the dialysis bag, 2% (v/v) of the supernatant was used to evaluate the algicidal activity on *A. tamarense*, instead of 1% v/v primitive supernatant of the BS01 culture. The results showed no algicidal effect was observed (data not shown). Therefore, it was speculated that the molecular weight of the algicidal compound was less than 500 Da. Several major fractions were collected after the silica column chromatography and Sephadex LH-20 gel chromatography analyses, and the TLC assays of the B_2_ faction from the second separation revealed two visual bands (see Supplementary Figure 1), one of which showed obvious algicidal activity and was named fraction C_2_. Figure 1A reveals that, at the 20 μg/mL concentration, fraction C_2_ exhibited a strong algicidal effect (64.5%) on *A. tamarense* while fraction C_1_ demonstrated almost no algicidal activity (8%). Therefore, fraction C_2_ was used for further analysis as the potential active compound. In addition, dimethyl sulphoxide (DMSO), known as alkahest, was used as a solvent for fraction C_2_ (insoluble in water) in this study, and no algicidal effects were shown (2%) in the DMSO treatments (negative controls), which is similar to the treatments with 2216E medium.

**Figure 2:**
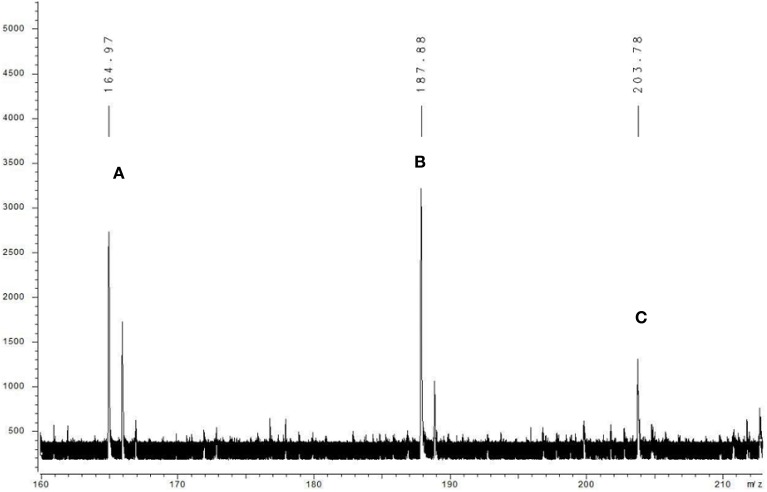
**Mass spectrum of fraction C_2_ obtained with MALDI-TOF MS**. The compound was dissolved in the carbon nanotube (CNT) matrix, and the data were recorded by positive ion mode. Letter A indicates the molecular ion peak, and B and C mark the positive ion peaks of [M+Na] ^+^ and [M+K]^+^, respectively.

**Figure 3:**
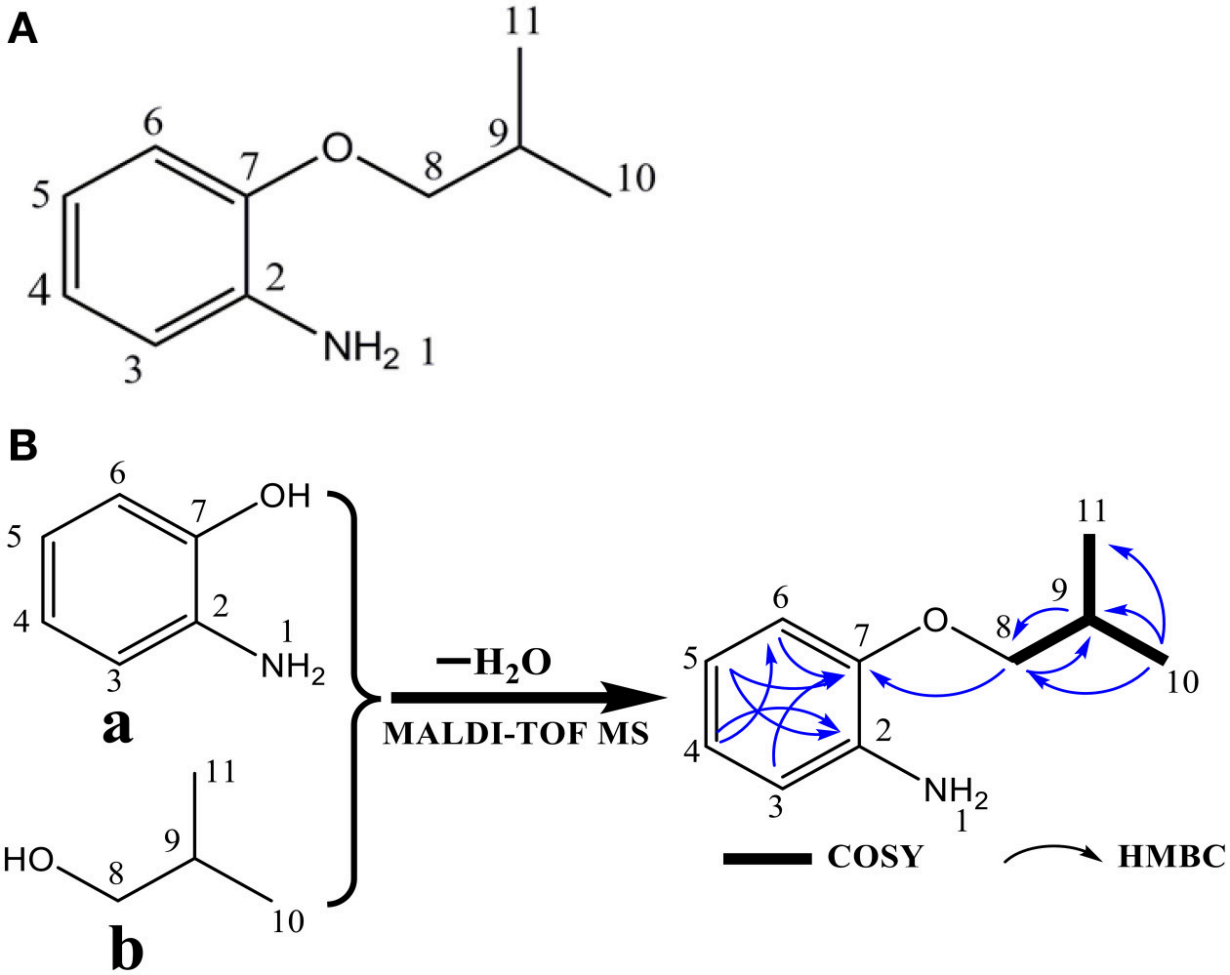
**Structure (A) and the fragments, key COSY, and HMBC correlations (B) of the (2-isobutoxyphenyl)amine algicidal compound**. In **(B)**, fragment a is 2-aminophenol, and fragment b is the 2-methylpropan-1-ol terminal residue.

The algicidal compound (fraction C_2_) was further identified by MS and NMR spectrum analysis. The MALDI-TOF MS spectrum revealed the pseudomolecular ions of fraction C_2_ at *m/z* of 187.88 [M+Na]^+^ and 203.78 [M+K]^+^ (Figure 2), and the ^1^H and ^13^C NMR spectra further suggested the molecular formula to be C_10_H_15_NO (Figure 3A). The linkage between C-2 and C-7 and the 1, 2-disubstituted phenyl group was suggested by the HMBC correlations of H-3 to C-7, H-5 to C-2 and C-7, and H-4 to C-2. The O and N atoms were preferentially and tentatively considered to be the substituents supported by the chemical shifts of C-2 and C-7, so the moiety was assigned as 2-aminophenol (Figure 3B-a). The 2-methylpropan-1-ol terminal residue fragment (Figure 3B-b) was suggested by the ^1^H-^1^H COSY correlation between H-8 and H-9 and H-9 and H-10/H-11 and the key HMBC correlations of H-10/H-11 to C-8 and C-9 (Supplementary Figures 2–7). The interpretation of the DEPT and ^1^H-^13^C HSQC spectra revealed the presence of two equivalent methyl groups at δ 1.11 (d, 3H, CH_3_-10 and CH_3_-11); one aliphatic methylene group at δ 3.81 (d, 2H, CH_2_-8), in which the chemical shift further indicated the linkage of the O atom to C-8; and five methine groups including one aliphatic methine group at δ 2.22 (m, 1H, CH-9) and four olefinicmethine groups at δ 6.76 (d, *J* = 7.4, 1.6, 1H, CH-3), 6.84 (t, 7.8, *J* = 1.7 Hz, 1H, CH-4), 6.82 (t, 7.8, *J* = 1.7 Hz, 1H, CH-5) and 6.78 (d, *J* = 7.6, 1.1, 1H, CH-6) (Supplementary Figures 8–14). Furthermore, the H-atoms showed that the AA'BB' system was ascribed to a 1, 2-disubstituted phenyl group with two substituted aromatic carbons at δ 136.4 (C-2) and δ 146.8 (C-7).

The SciFinder search showed that the chemical structure of the substance purified from this study matched that of (2-isobutoxyphenyl)amine (CAS No. 104065-95-4, a type of aromatic amine) with the identical molecular formula of C_10_H_15_NO (Figures 3A,B). The TLC assays using the mixture of ethyl acetate and methanol as a developing solvent also provided evidence that (2-isobutoxyphenyl)amine and the purified substance (fraction C_2_) were affiliated with the same chemical family with an identical RFs value (data not shown), further demonstrating that the purified compound and (2-isobutoxyphenyl)amine were the same chemical substance.

### Determination of algicidal compound

HPLC analysis was used to detect the algicidal compound in the BS01 culture by observing the retention time. In the supplementary information, Supplementary Figure 15 illustrated chromatograms of (2-isobutoxyphenyl)amine) of the standard sample and algicidal culture. In the chromatogram of the standard, a distinct peak was observed at the retention time of 11.27 min. Similarly, at the same retention time, a relatively small peak was detected in the algicidal culture of BS01. Owing to the complex compounds in the algicidal culture, many other peaks were detected under the ultraviolet wavelength of 288 nm.

### Biological activity of (2-isobutoxyphenyl)amine against *A. tamarense*

Commercial (2-isobutoxyphenyl)amine (> 98% purity) was dissolved in DMSO and used for an algicidal activity assay. Figure 1B illustrated the algicidal effects of commercial (2-isobutoxyphenyl)amine at different concentrations (5, 10, 15, and 20 μg/mL) against *A. tamarense* over time (4, 8, 12, and 24 h), and a One-Way ANOVA revealed significant differences between the treatments and their corresponding controls. Greater algicidal activity on *A. tamarense* was observed with higher concentrations of (2-isobutoxyphenyl)amine and longer treatment times. At 4 h under the (2-isobutoxyphenyl)amine treatment, there was significant algicidal activity with the less than 60,000/mL concentration of algal cells (*p* < 0.05). After treatment with (2-isobutoxyphenyl)amine for 8 and 12 h, the numbers of cells in the treatments were much less than in the controls, less than 50,000 in 1 mL of algal culture. Under the 20 μg/mL (2-isobutoxyphenyl)amine treatment for 24 h, the algicidal activity was 56.5 and 59.7% compared with the 2216E and DMSO treatments, respectively.

**Figure 4:**
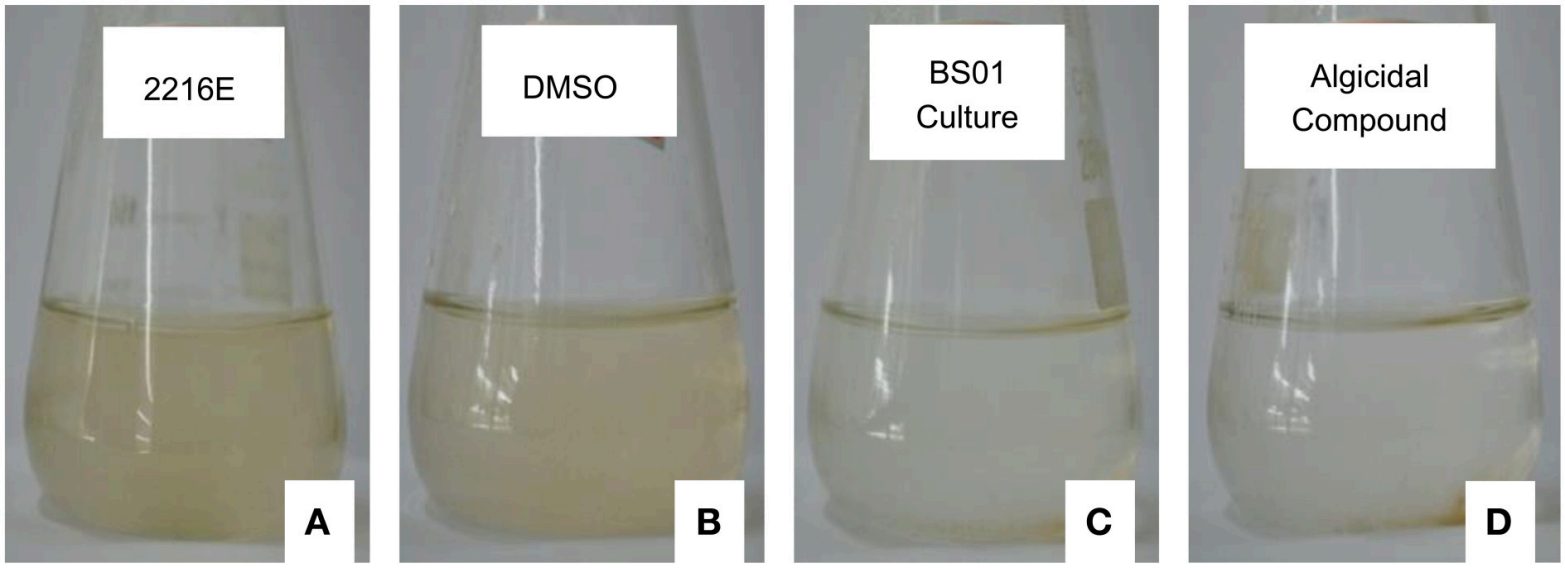
**Algal culture growth treated by the standard compound and bacterial culture (1%, v/v) for 24 h. (A,C)** Show the algal culture with the addition of 1% (v/v) of sterile Zobell 2216E and the BS01 culture, respectively. **(B,D)** depict the algal culture treated with the same volume of DMSO and 20 μg/mL of (2-isobutoxyphenyl)amine, respectively.

As shown in Figure 4, algal cells aggregated and were deposited at the bottom of the flasks when treated with 20 μg/mL of (2-isobutoxyphenyl)amine, and based on observations under the light microscope (10 × 40), the mobility of the cells was lost at 24 h. Similarly, the loss of cellular integrity and mobility was also observed under the treatment with 1% of BS01 culture (v/v); the color of the algal culture became green and then ivory. However, the algal cells in the controls, which were treated by Zobell 2216E and DMSO, remained active and suspended in f/2 medium. Under the light microscope, the algal cells in the controls were observed moving freely.

**Figure 5:**
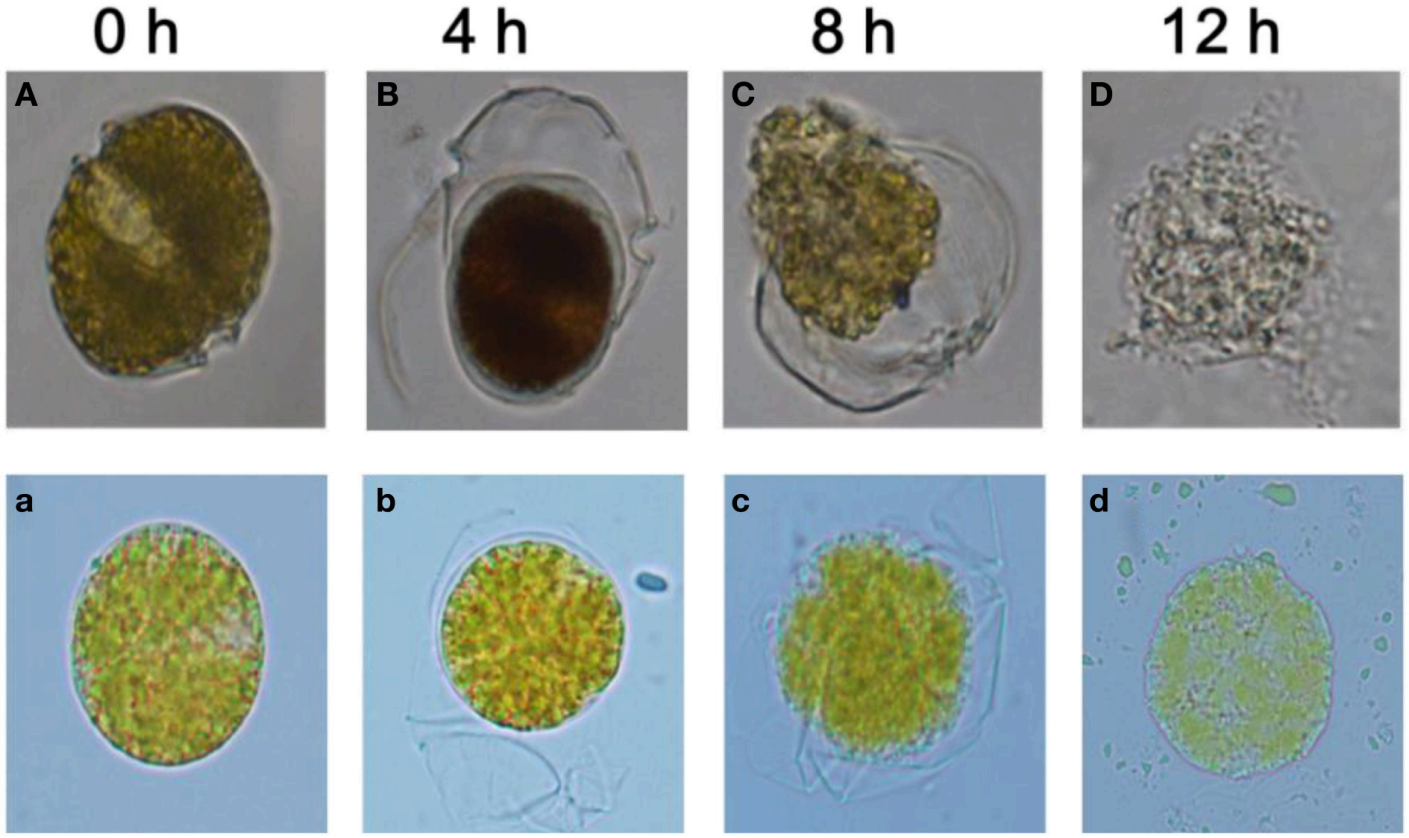
**Morphological changes in *A. tamarense* after exposure to BS01 culture and 20 μg/mL of (2-isobutoxyphenyl)amine (0~12 h) under an optical microscope (10 × 100)**. **(A)** Control with the addition of the same volume of 2216E; **(B–D)** algal cells treated with 1% (v/v) of the BS01 supernatant; **(b–d)** cells treated with 20 μg/mL of (2-isobutoxyphenyl)amine; **(a)** control with the addition of the same amount of DMSO.

Morphological analysis revealed structural alterations in *A. tamarense* with algal cells losing the integrity of their organelles (Figure 5). Compared with the control cells in Figure 5A (with exposure to DMSO or 2216E), these treated cells exhibited many morphological differences and even structural damage (Figures 5B–D and Figures 5b–d). In the control cells, the cellular structures were intact with two distinct carapaces and dense cytoplasm, but in the treatments with the 1% (v/v) BS01 supernatants or (2-isobutoxyphenyl)amine (20 μg/mL), the structural integrity of the algal cells was lost, and plasmolysis and vacuolization, and even degradation, of the cytoplasm were observed. *A. tamarense* cells treated with the 1.0% BS01 supernatant for 4 h showed further concentration of the cytoplasm with the separation of the cell walls (Figure 5B). With extended exposure (8 h), the cytoplasm became completely divorced from the cell well and dispersed into the culture solution (Figure 5C). At 12 h, the cytoplasm began to degrade into small, irregular debris particles, and the algal cells thoroughly disintegrated. With (2-isobutoxyphenyl)amine, the treated cells displayed an obvious profile of plasmolysis and even vacuolization and eventually became debris (Figures 5b–d). However, it appeared that the integrity of the cells under the (2-isobutoxyphenyl)amine treatment remained, which contrasted with those treated by supernatants whose cellular debris diffused into the algal culture.

### Degradation of nuclear structure

**Figure 6:**
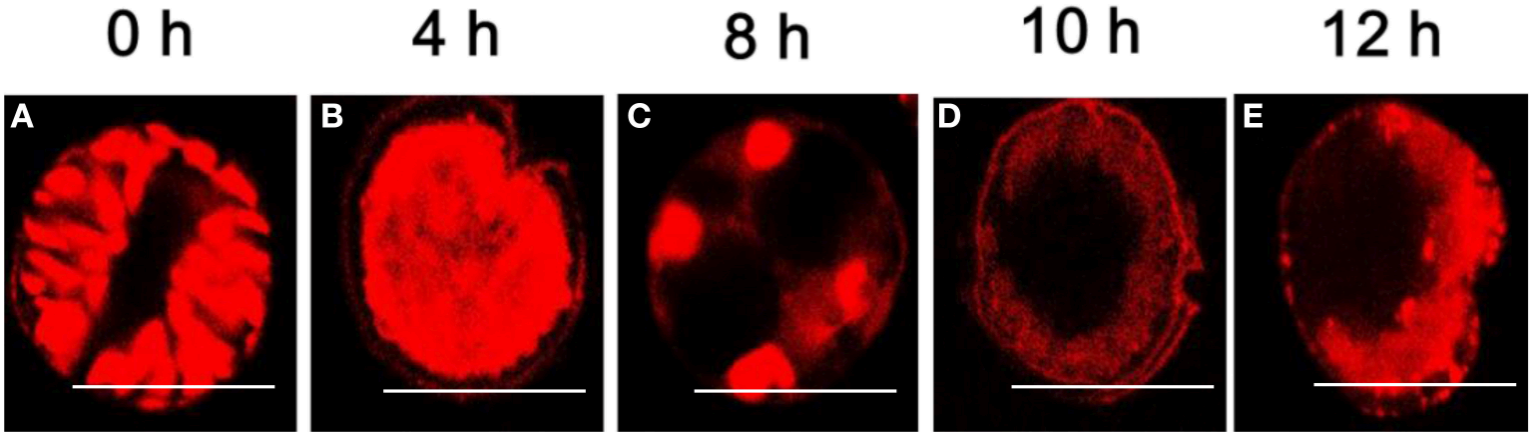
**Degradation of the nuclear structure of *A. tamarense* after exposure to 20 μg/mL of (2-isobutoxyphenyl)amine over time (0~12 h). (A)** Control cells (same volume of DMSO); **(B–E)** degradation of the nuclear structure of the algal cells after exposure to (2-isobutoxyphenyl)amine (20 μg/mL). Red fluorescence represents the nuclear area, and the scale bar = 20 μm. (For the interpretation of the color references in this figure, refer to the online version of this article).

PI can only pass through a dead cell membrane and emits red fluorescence after being embedded in double-stranded DNA, but in the control group, the entire algal cell emitted red fluorescence as a result of auto-fluorescence (Figure 6A). When exposed to (2-isobutoxyphenyl)amine for 4 h, the algal cells were gradually concentrated with intense red fluorescence (Figure 6B), which means that PI passed through the membrane of the dead cell because it was permeable. At 8 h, dramatic changes were observed; the cell nucleoplasm exhibited compact punctate organelles with red fluorescence and migrated to the edge of the cells (Figure 6C). Subsequently, there was distinct degradation characterized by empty regions and the lack of recognizable organelles while the cell membranes remained intact (Figure 6D). Late in the treatment, most of the treated cells were characterized by more dramatic vacuolization and internal degradation and appeared empty, as shown in Figure 6E.

## Discussion

In the previous study, the BS01 bacterium indirectly exerted a strong algicidal effect on *A. tamarense* by secreting stable compounds (Bai et al., 2011), and the filters exhibited algicidal activities even after water bath treatments (up to 100°C for 30 min) and enzymatic digestion (Proteinase K at a final concentration 0.05 mg/mL at 65°C for 1 h), indicating that the algicidal compound(s) cannot be enzymatic. Hence, in this study, filtrate was directly used to isolate the algicidal compounds and proteins were excluded without further analysis. The BS01 supernatant extract could be easily extracted by ethyl acetate, indicating that the algicidal substance, in addition to being non-proteinaceous, was hydrophobic with moderate polarity, so Sephadex LH-20 and silica gel were applied to purify the compound. MS and NMR analysis identified the active algicidal component isolated from the BS01 culture as (2-isobutoxyphenyl)amine, which is an aniline compound, making this the first report of this substance as an algicide. However, the compound has been previously reported as a material used in the synthesis of anti-allergens (Ozeki et al., 1989), anticarcinogens (Huang et al., 2011), and antibiotics (El-Gendy et al., 2008). In this study, HPLC analysis was used to further verify whether (2-isobutoxyphenyl)amine was excreted in the culture of BS01. According to the characteristics of molecular polarity, different compounds were eluted and detected at their certain retention times (Kawasaki et al., 1986). (2-isobutoxyphenyl)amine was detected at the retention time of 11.27 min in the standard and algicidal culture, elucidating (2-isobutoxyphenyl)amine was the algicidal compound and excreted in the BS01 culture.

Substances that have been previously reported to inhibit the growth of algae are commonly isolated from bacteria in the γ*-proteobacteria* phylum, which includes the genera *Alteromonas* (Dakhama et al., 1993), *Pseudoalteromonas* (Gregory et al., 1999), and *Vibrio* (Ichiro and Satoshi, 2008). Nakashima et al. (2006) reported that the γ*-proteobacterium* MS-02-063 displayed potent algicidal activity against various red tide phytoplanktons by secreting PG-L-1 pigment in a concentration-dependent manner. Ichiro and Satoshi (2008) isolated one strain of *Pseudoalteromonas*, which can kill the dinoflagellate *Cochlodinium polykrikoides*. Nevertheless, BS01 had a strong algicidal effect on the dinoflagellate *A. tamarense* and was closely related to the *Brevibacterium* genus of the Actinomycetales (Bai et al., 2011), which was the first such report for this group. Furthermore, the algicidal compounds produced by the marine bacterium BS01 were considered to be heat tolerant and stable in acidic or alkali conditions, and they exhibited a wide range of algicidal activities (Bai et al., 2011). These results implied that there might be a great abundance of microorganism playing a vital role in the control of algal growth.

The previous laboratory study reported that a 24-h BS01 culture broth following the fractionation treatment of all molecular weight cut-off dialysis bags (100~3000) did not exhibit any algicidal activity, which indicates that the molecular weight of the algicidal compound(s) might be less than 100 (Bai et al., 2011). However, in this study, the algicidal compound was purified and identified as having a molecular weight of 165. These two paradoxical results may be explained by a hypothesis that different species of secondary metabolites accumulate during different growth phages (24 and 48 h). This study focused on the algicidal compounds produced in the stationary phase (48 h) when the secondary metabolite, (2-isobutoxyphenyl)amine, accumulated to some extent and exerted its algicidal effect on *A. tamarense*. It can be concluded that the algicidal activity of these compounds was not only concentration-dependent but also time-dependent.

To evaluate the characteristics of the algicidal extracts of strain BS01, we studied the influence of (2-isobutoxyphenyl)amine on *A. tamarense* under different treatment concentrations and exposure times. Treatment with 20 μg/mL of (2-isobutoxyphenyl)amine could lead to an algal death rate of 59.7% at 24 h compared to a treatment with an equal volume of DMSO. The validation of algicidal activity revealed that the effects of the algicidal extracts (Fraction C_2_) were strong (64.5%) on *A. tamarense* at a concentration of 20 μg/mL. There have been several other reports on the properties of algicidal compounds under different concentrations with treatment time. Yoshikawa et al. (2000) found that some cyanobacteria were sensitive to L-CNAla at a concentration of 0.4–25 mg/mL, and Seunghak et al. (2003) reported that 20 mg/L of sophorolipid was an effective concentration for the mitigation of HABs. Kim et al. (2008) observed significant algicidal activity in prodigiosin from *Hahella chejuensis* against *Cochlodinum polykridoides* at a concentration of 4–10 mg/L. Therefore, (2-isobutoxyphenyl)amine is not the most efficient compound that has been reported, but it showed high efficiency in the inhibition of *A. tamarense* growth, thus adding an important compound to the algicidal substance library. In addition, in this study, it seemed that the algicidal culture exhibited a stronger algicidal effect on *A. tamarense* than (2-isobutoxyphenyl)amine. It was presumed other types of bioactive materials likely persisted in the BS01 culture after fermentation, which interacted with each other and had synergistic effects on *A. tamarense*.

The cell membrane is a barrier that prevents extracellular substances from passing freely through algal cells; thus it maintains a stable environment in the cells so that the various biochemical reactions can be operated (Veldhuis et al., 2010). There have been several reports that bacterial cultures could change the permeability of the cell membrane and break its integrity, inducing the algal cells to lose their intracellular materials (Li et al., 2014). Several types of morphological changes have taken place in algal cells treated with different types of algicidal compounds (Zhang et al., 2013; Li et al., 2014). The volatile algicidal compounds from cyanobacteria caused shrinking and then wrinkling, and direct contact with terpenoids resulted in stripping while swelling and then collapse could be caused by basic amino-acids (Ozaki et al., 2009). Our result showed the *A. tamarense* cells initially shrank and then exhibited plasmolysis and even vacuolization, eventually creating debris with the loss of cell integrity. When subject to the algicidal supernatant from the marine algicidal actinomycete BS01, the cytoplasm began to degrade into small irregular debris particles, and the algal cells finally became thoroughly disassembled. The resulting differences in final morphology can likely be ascribed to the different roles of several of the bioactive materials from BS01 in the process of algal death. During the death of *A. tamarense*, the color of the algal culture changed from green to ivory, which might be due to the algal cells breaking and the contents (such as mitochondria, chloroplasts and nuclei) leaking out. The chloroplasts could be particularly damaged as chlorophyll a degrades into phaeophorbide ester (Berger et al., 1979; Rice, 1984; Li and Hu, 2004; Bidle et al., 2007).

The change in nuclear structure under red fluorescence could be observed in the algal cells stained by propidium iodide under the algicide treatment over a period of time. We observed that the nucleus became highly condensed in a short time when the algal cells were treated with (2-isobutoxyphenyl)amine, but the cellular nuclear gradually degraded and eventually disappeared over an extended treatment time. Li et al. (2014) reported that the nuclear membrane might break, and the nuclear material might disperse in algal cells when subjected to algicidal extracts. The results implied that the nucleus was influenced by the algicidal compound and the nuclear structure was damaged, so the nucleus might be the primary target of (2-isobutoxyphenyl)amine.

## Conclusions

In conclusion, (2-isobutoxyphenyl)amine, isolated and identified from BS01 culture, exerted a strong algicidal effect on *A. tamarense*. The molecular mass of this algicidal compound was 165 Da with a molecular formula of C_10_H_15_NO. The results revealed that algal movements were inhibited when cells were exposed to (2-isobutoxyphenyl)amine, and the algicidal fraction destroyed the cell membrane integrity and damaged the nuclear structure. With its high and novel algicidal activity, (2-isobutoxyphenyl)amine provides the possibility of controlling *A. tamarense* blooms through a microbial strategy and extends the resource pool of algicidal compounds for use against harmful algal blooms. Furthermore, Based on the fact that (2-isobutoxyphenyl)amine is not solely responsible for BS01's algicidal effects and there could be more effective unidentified compounds in the bioactive fraction. Therefore, we suggest that the better way is to use bacterial agent (produced by BS01) instead of its algicidal agent [(2-isobutoxyphenyl)amine] when practicing the control of HAB causing by *A. tamarense*.

### Conflict of interest statement

The authors declare that the research was conducted in the absence of any commercial or financial relationships that could be construed as a potential conflict of interest.

## Supplementary material

Supplementary Figure 1 **Thin-layer chromatograph analysis of fraction B_1_ and B_2_ using iodine as a chromogenic solvent and the mixture of ethyl acetate and methanol as developing solvent**. The number 1 represents for fraction B_1_ and number 2 stands for fraction B_1_ from the second chromatography separation. In the TLC analysis of fraction B_2_, fraction C_2_ with the purple red-like color was separated, which showed strong algicdal effect on *A. tamarense*.

Supplementary Figure 2 **Heteronuclear multibond correlations (HMBC) spectrum (^2^J_CH_, ^3^J_CH_, 600 MHz) of fraction C_2_ in CDCl_3_ (^13^C: 0.5–9 ppm, ^1^H: 0–180 ppm)**.

Supplementary Figure 3 **Heteronuclear multibond correlations (HMBC) spectrum (^2^J_CH_, ^3^J_CH_, 600 MHz) of fraction C_2_ in CDCl_3_ (^13^C: 7.35–7.9 ppm, ^1^H: 10–83 ppm)**.

Supplementary Figure 4 **Heteronuclear multibond correlations (HMBC) spectrum (^2^J_CH_, ^3^J_CH_, 600 MHz) of fraction C_2_ in CDCl_3_ (^13^C: 0.7–4.5 ppm, ^1^H: 125–170 ppm)**.

Supplementary Figure 5 **^1^H–^1^H chemical shift correlation spectroscopy (COSY) spectrum (600 MHz) of fraction C_2_ in CDCl_3_ (horizontal axis: 0.5–8.0 ppm, vertical axis: 0.5–8 ppm)**.

Supplementary Figure 6 **^1^H–^1^H chemical shift correlation spectroscopy (COSY) spectrum (600 MHz) of fraction C_2_ in CDCl_3_ (horizontal axis: 7.05–7.9 ppm, vertical axis: 7.05–8 ppm)**.

Supplementary Figure 7 **^1^H–^1^H chemical shift correlation spectroscopy (COSY) spectrum (600 MHz) of fraction C_2_ in CDCl_3_ (horizontal axis: 0.67–2.46 ppm, vertical axis: 0.5–4.6 ppm)**.

Supplementary Figure 8 **Distortionless Enhancement by Polarization Transfer (DEPT) spectrum of fraction C_2_ in CDCl_3_ (chemical shift: 11–174 ppm)**.

Supplementary Figure 9 **Distortionless Enhancement by Polarization Transfer (DEPT) spectrum of fraction C_2_ in CDCl_3_ (chemical shift: 60–140 ppm)**.

Supplementary Figure 10 **Distortionless Enhancement by Polarization Transfer (DEPT) spectrum of fraction C_2_ in CDCl_3_ (chemical shift: 5–60 ppm)**.

Supplementary Figure 11 **^1^H NMR spectrum (600 MHz) of fraction C_2_ in CDCl_3_ (chemical shift: 0.7–7.9 ppm)**.

Supplementary Figure 12 **^1^H NMR spectrum (600 MHz) of fraction C_2_ in CDCl_3_ (chemical shift: 0.7-4.5 ppm)**.

Supplementary Figure 13 **Nuclear overhauser enhancement spectroscopy (NOESY) (600 MHz) of fraction C_2_ in CDCl_3_**.

Supplementary Figure 14 **Heteronuclear single quantum correlation (HSQC) spectrum (600 MHz) of fraction C_2_ in CDCl_3_**.

Supplementary Figure 15 **HPLC analysis of the authentic standard ((2-isobutoxyphenyl)amine, 5 ppm) and the algicidal culture of BS01**. The algicidal culture was prepared by extracting 200 mL culture for three times with equal volumes of ethyl acetate.
