# Supplementary figures and images for: Discovery of an algicidal compound from Brevibacterium sp. BS01 and its effect on a harmful algal bloom-causing species, Alexandrium tamarense
Source: Front Microbiol. 2015 Nov 5;6:1235. doi: 10.3389/fmicb.2015.01235 (PMC4633486; doi:10.3389/fmicb.2015.01235)

AXL-BC

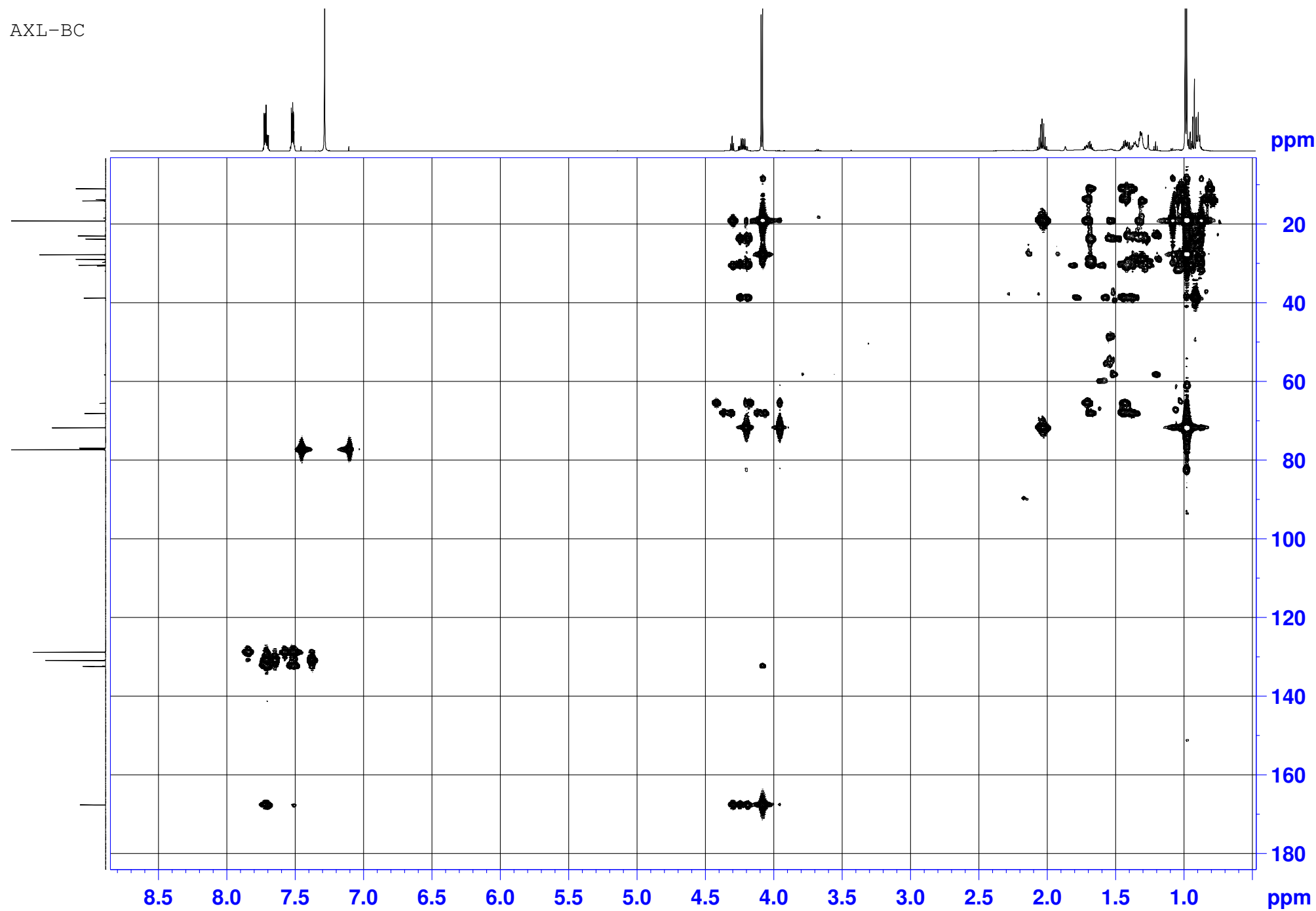

Supplement: Supplementary Figure 1 — Thin-layer chromatograph analysis of fraction B1 and B2 using iodine as a chromogenic solvent and the mixture of ethyl acetate and methanol as developing solvent. The number 1 represents for fraction B1 and number 2 stands for fraction B1 from the second chromatography separation. In the TLC analysis of fraction B2, fraction C2 with the purple red-like color was separated, which showed strong algicdal effect on A. tamarense. [file Image1.PDF]

AXL-BC

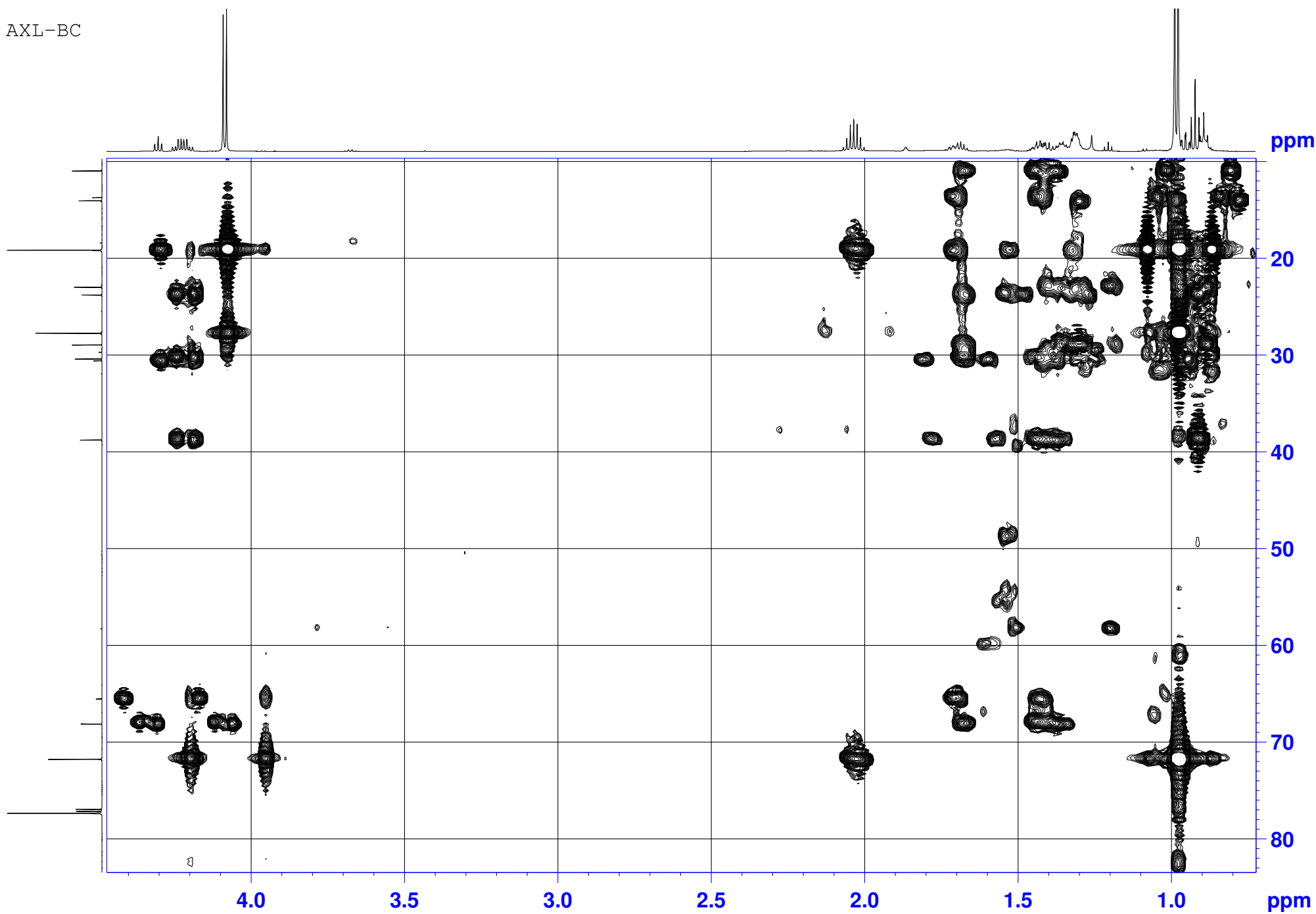

Supplement: Supplementary Figure 2 — Heteronuclear multibond correlations (HMBC) spectrum (2JCH, 3JCH, 600 MHz) of fraction C2 in CDCl3 (13C: 0.5–9 ppm, 1H: 0–180 ppm). [file Image2.PDF]

AXL-BC

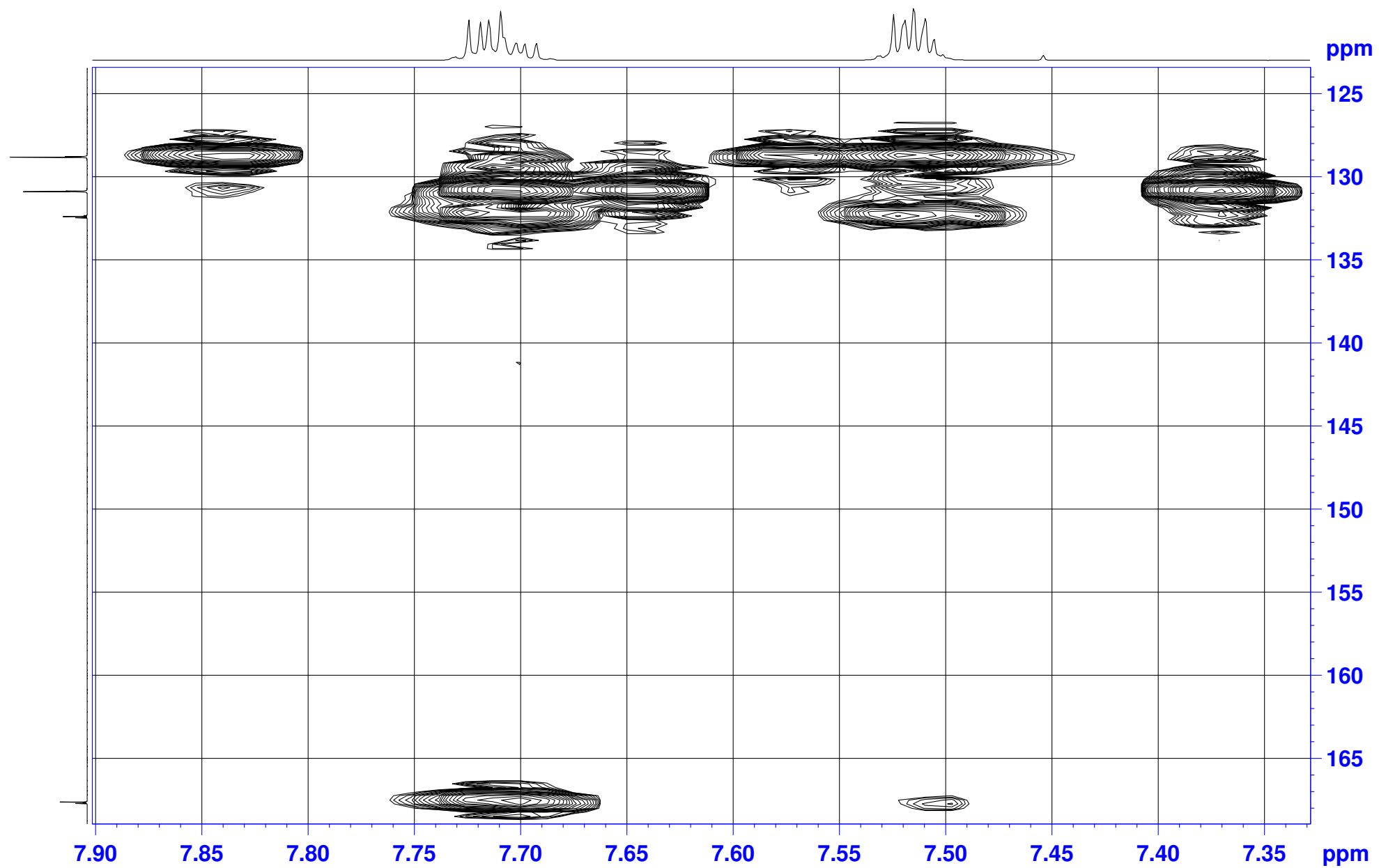

Supplement: Supplementary Figure 3 — Heteronuclear multibond correlations (HMBC) spectrum (2JCH, 3JCH, 600 MHz) of fraction C2 in CDCl3 (13C: 7.35–7.9 ppm, 1H: 10–83 ppm). [file Image3.PDF]

AXL-COSY

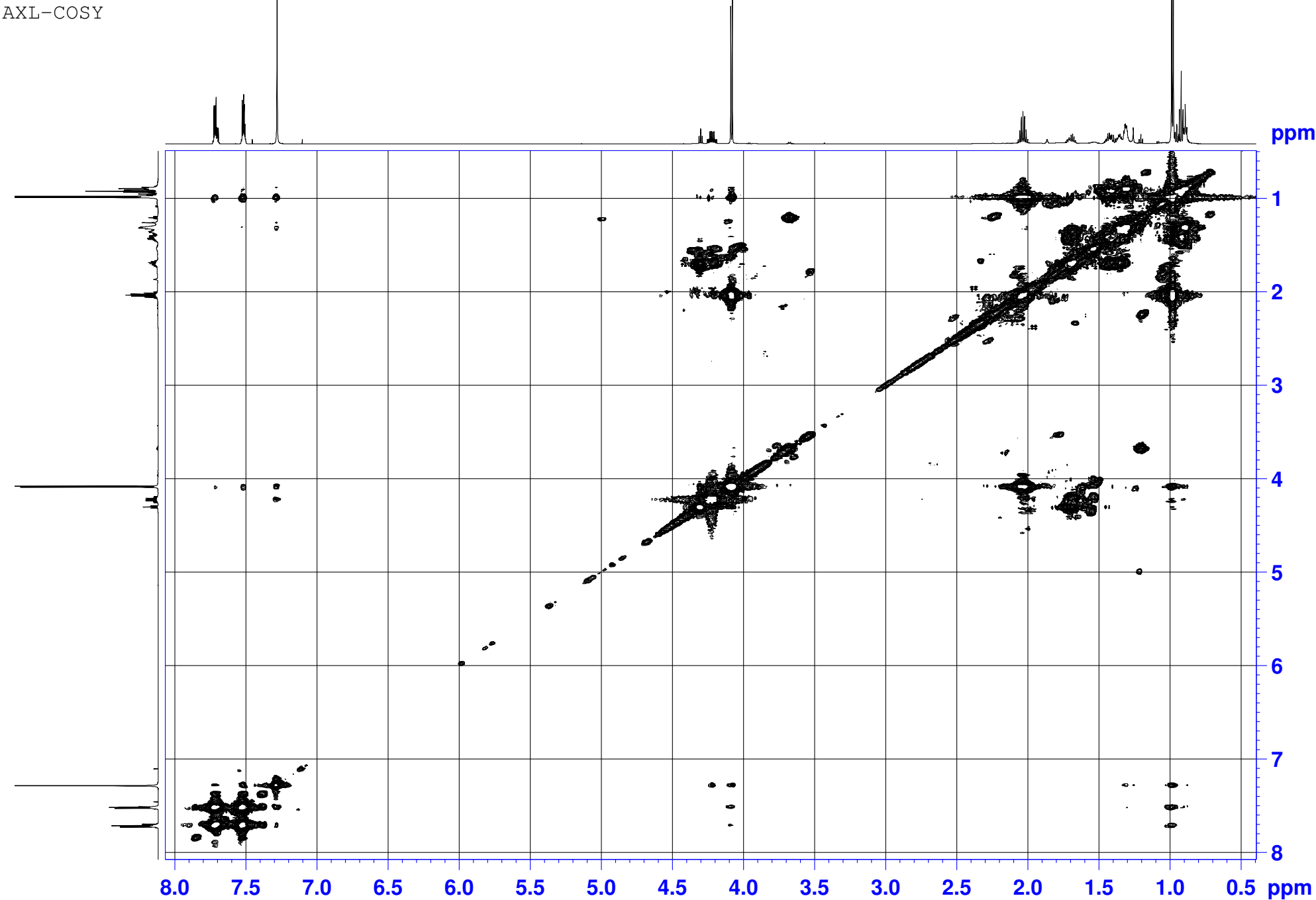

Supplement: Supplementary Figure 4 — Heteronuclear multibond correlations (HMBC) spectrum (2JCH, 3JCH, 600 MHz) of fraction C2 in CDCl3 (13C: 0.7–4.5 ppm, 1H: 125–170 ppm). [file Image4.PDF]

AXL-COSY

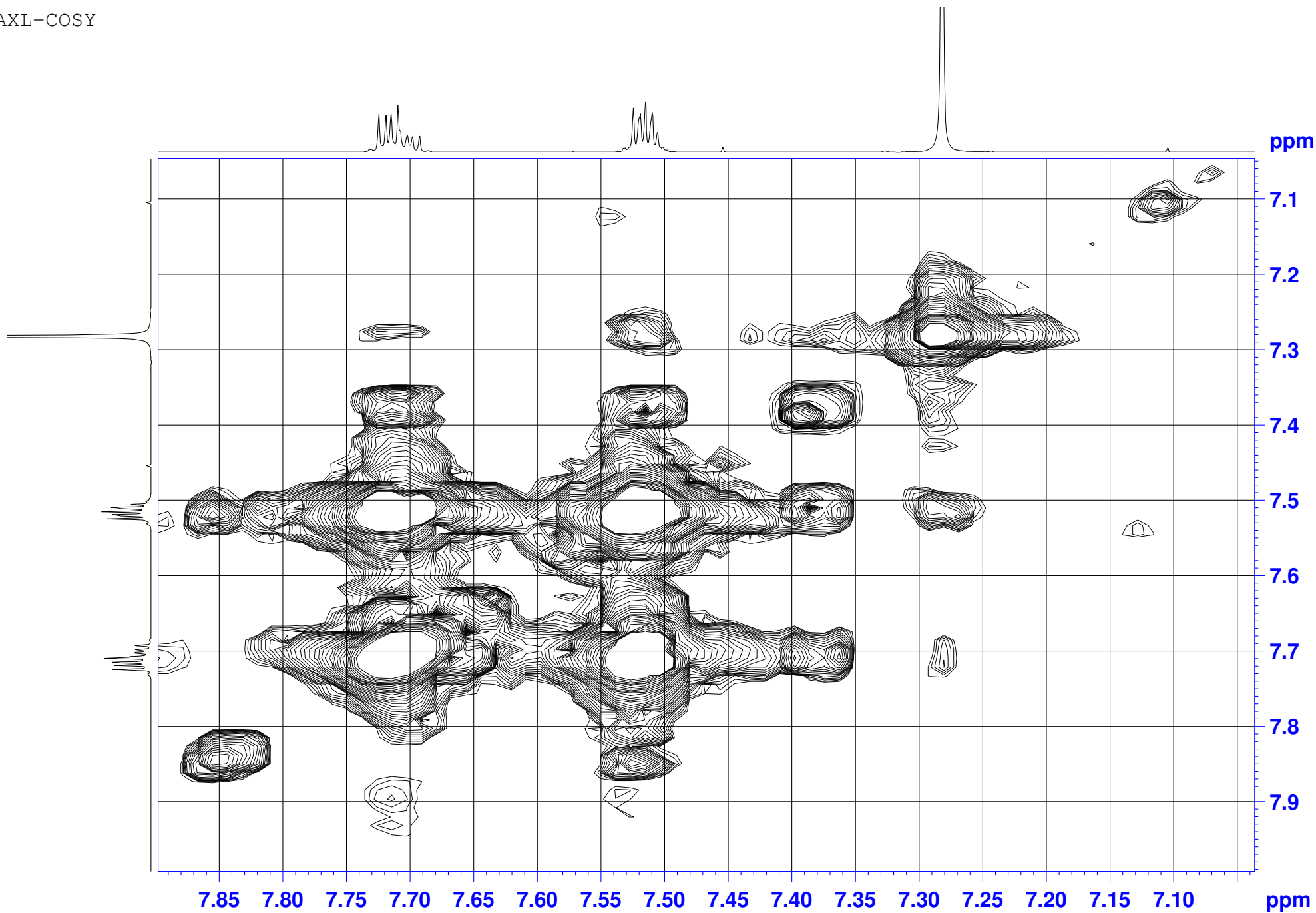

Supplement: Supplementary Figure 5 — 1H–1H chemical shift correlation spectroscopy (COSY) spectrum (600 MHz) of fraction C2 in CDCl3 (horizontal axis: 0.5–8.0 ppm, vertical axis: 0.5–8 ppm). [file Image5.PDF]

AXL-COSY

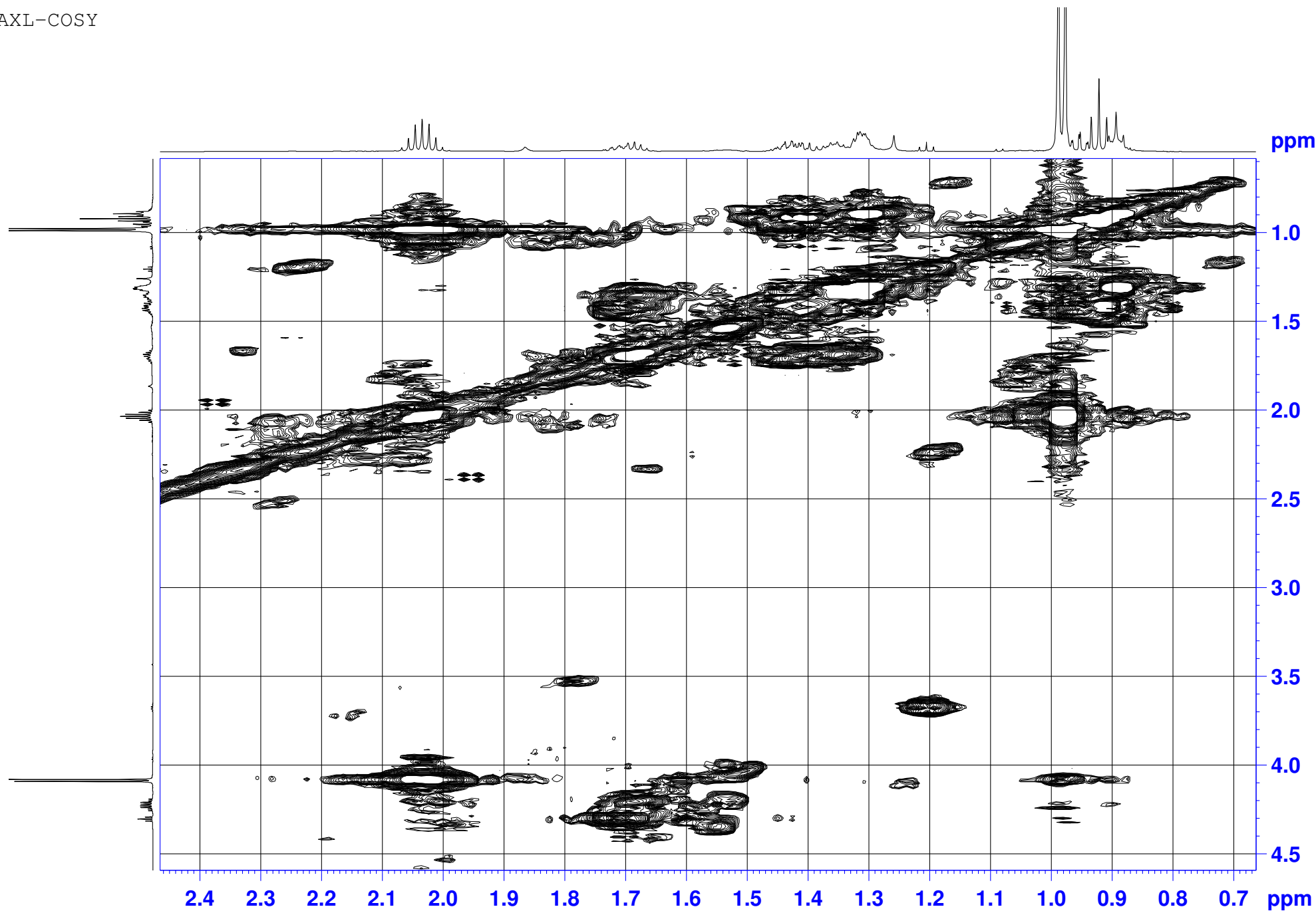

Supplement: Supplementary Figure 6 — 1H–1H chemical shift correlation spectroscopy (COSY) spectrum (600 MHz) of fraction C2 in CDCl3 (horizontal axis: 7.05–7.9 ppm, vertical axis: 7.05–8 ppm). [file Image6.PDF]

AXL-H

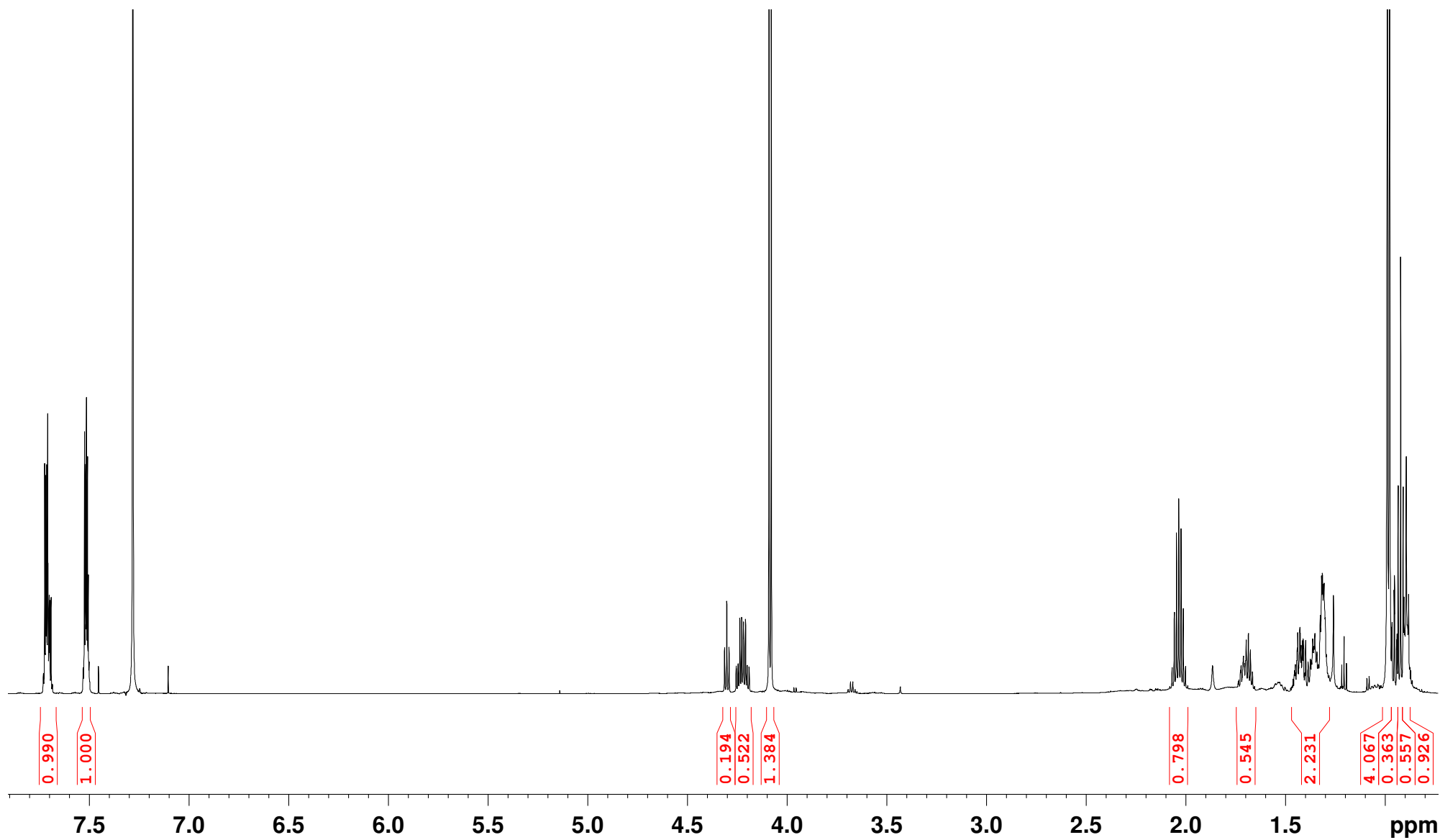

Supplement: Supplementary Figure 10 — Distortionless Enhancement by Polarization Transfer (DEPT) spectrum of fraction C2 in CDCl3 (chemical shift: 5–60 ppm). [file Image10.PDF]

AXL-H

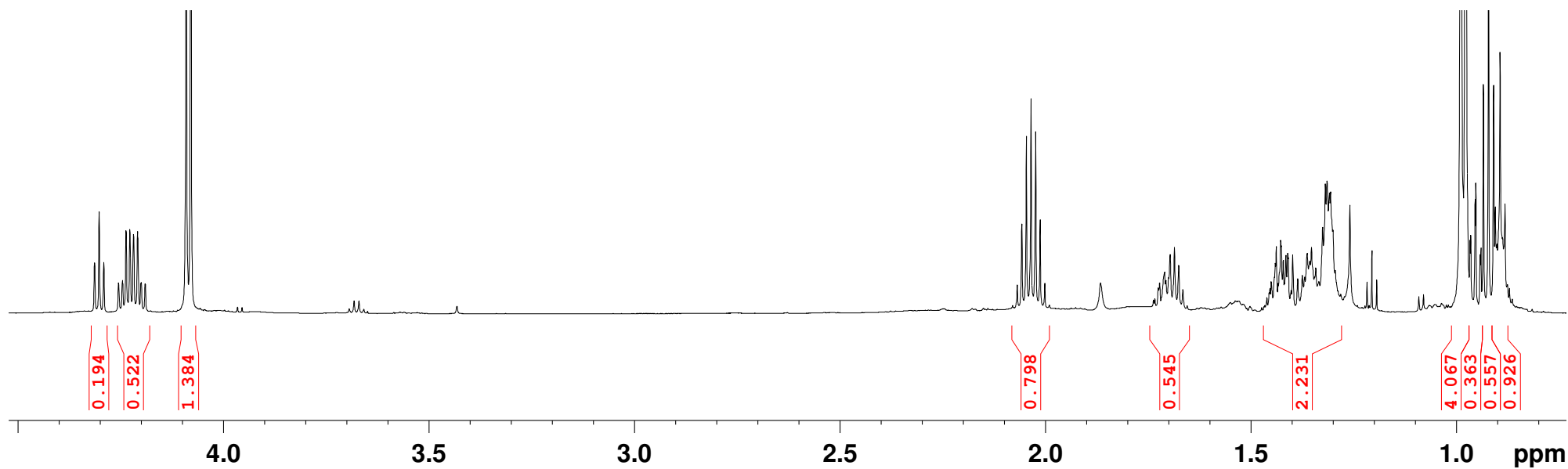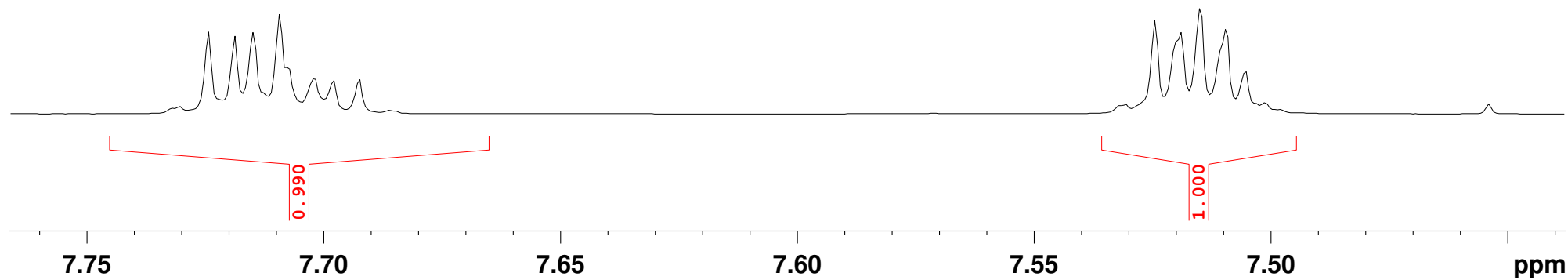

Supplement: Supplementary Figure 11 — 1H NMR spectrum (600 MHz) of fraction C2 in CDCl3 (chemical shift: 0.7–7.9 ppm). [file Image11.PDF]

AXL-NOE

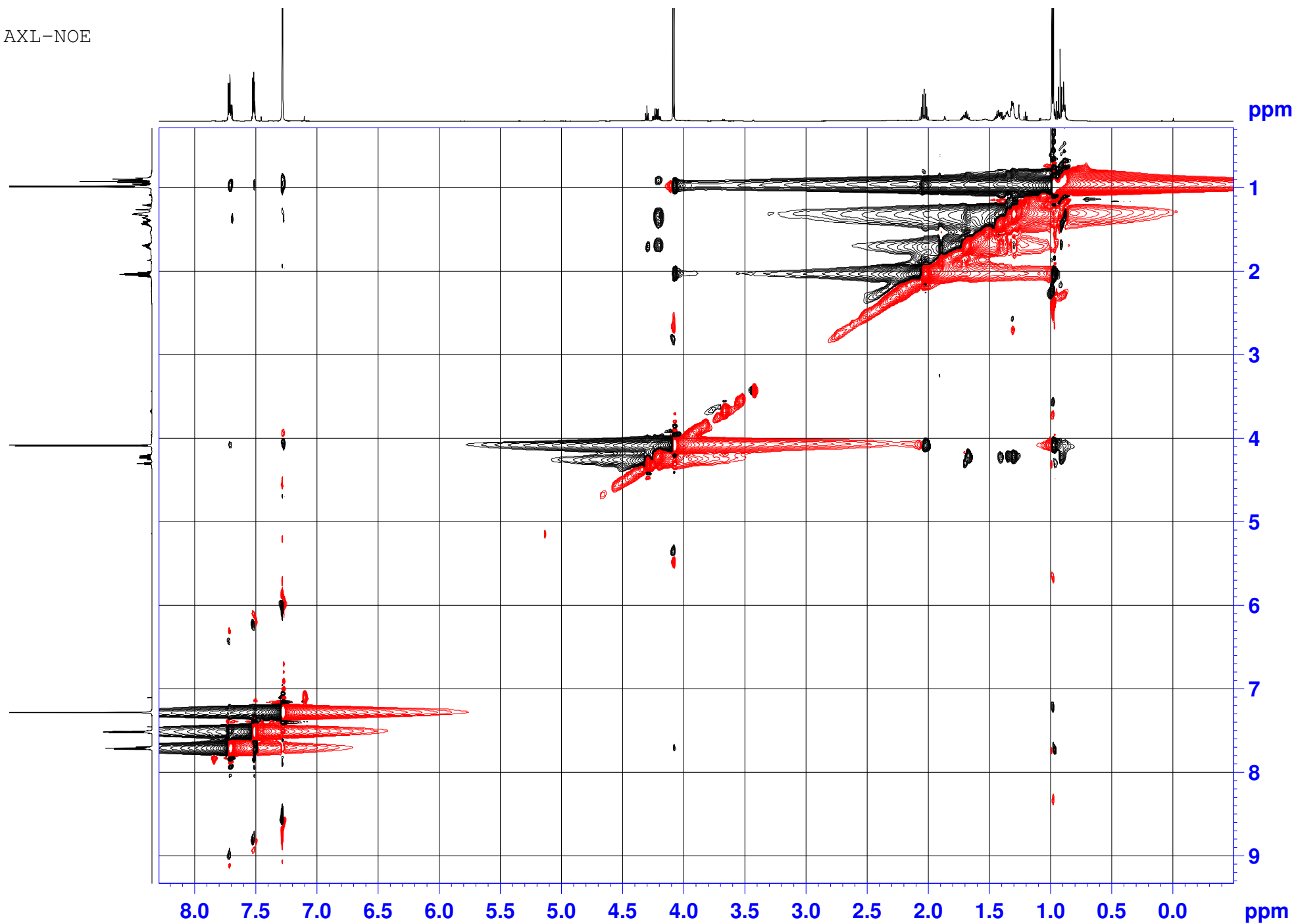

Supplement: Supplementary Figure 12 — 1H NMR spectrum (600 MHz) of fraction C2 in CDCl3 (chemical shift: 0.7-4.5 ppm). [file Image12.PDF]

AXL-QC

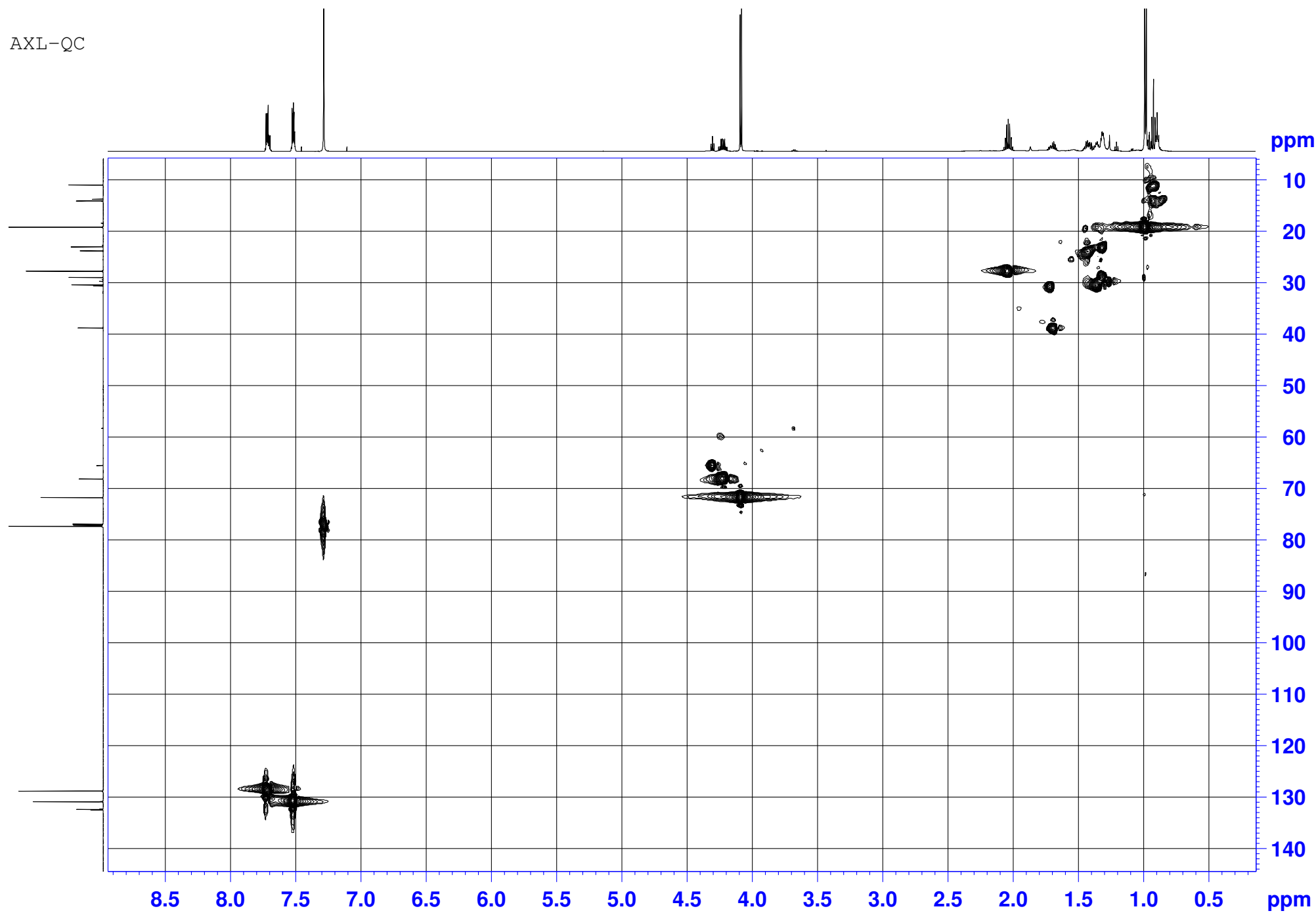

Supplement: Supplementary Figure 13 — Nuclear overhauser enhancement spectroscopy (NOESY) (600 MHz) of fraction C2 in CDCl3. [file Image13.PDF]

AXL-QC

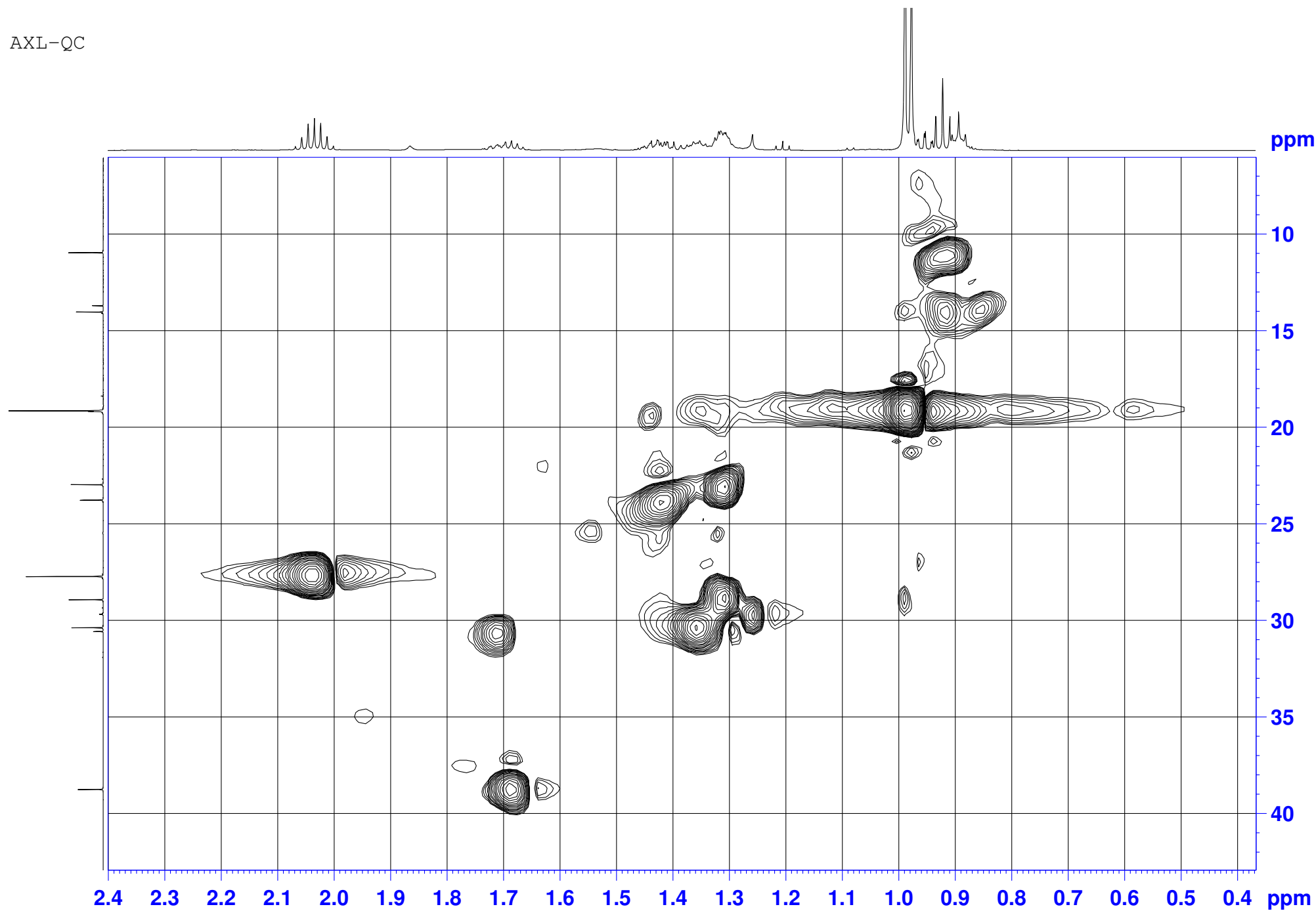

Supplement: Supplementary Figure 14 — Heteronuclear single quantum correlation (HSQC) spectrum (600 MHz) of fraction C2 in CDCl3. [file Image14.PDF]
