# Supplementary material for: Discovery of an algicidal compound from Brevibacterium sp. BS01 and its effect on a harmful algal bloom-causing species, Alexandrium tamarense
Source: Front Microbiol. 2015 Nov 5;6:1235. doi: 10.3389/fmicb.2015.01235 (PMC4633486; doi:10.3389/fmicb.2015.01235)
Supplement: Supplementary Figure 7 — 1H–1H chemical shift correlation spectroscopy (COSY) spectrum (600 MHz) of fraction C2 in CDCl3 (horizontal axis: 0.67–2.46 ppm, vertical axis: 0.5–4.6 ppm). [file Image7.PDF]

AXL-DEPT

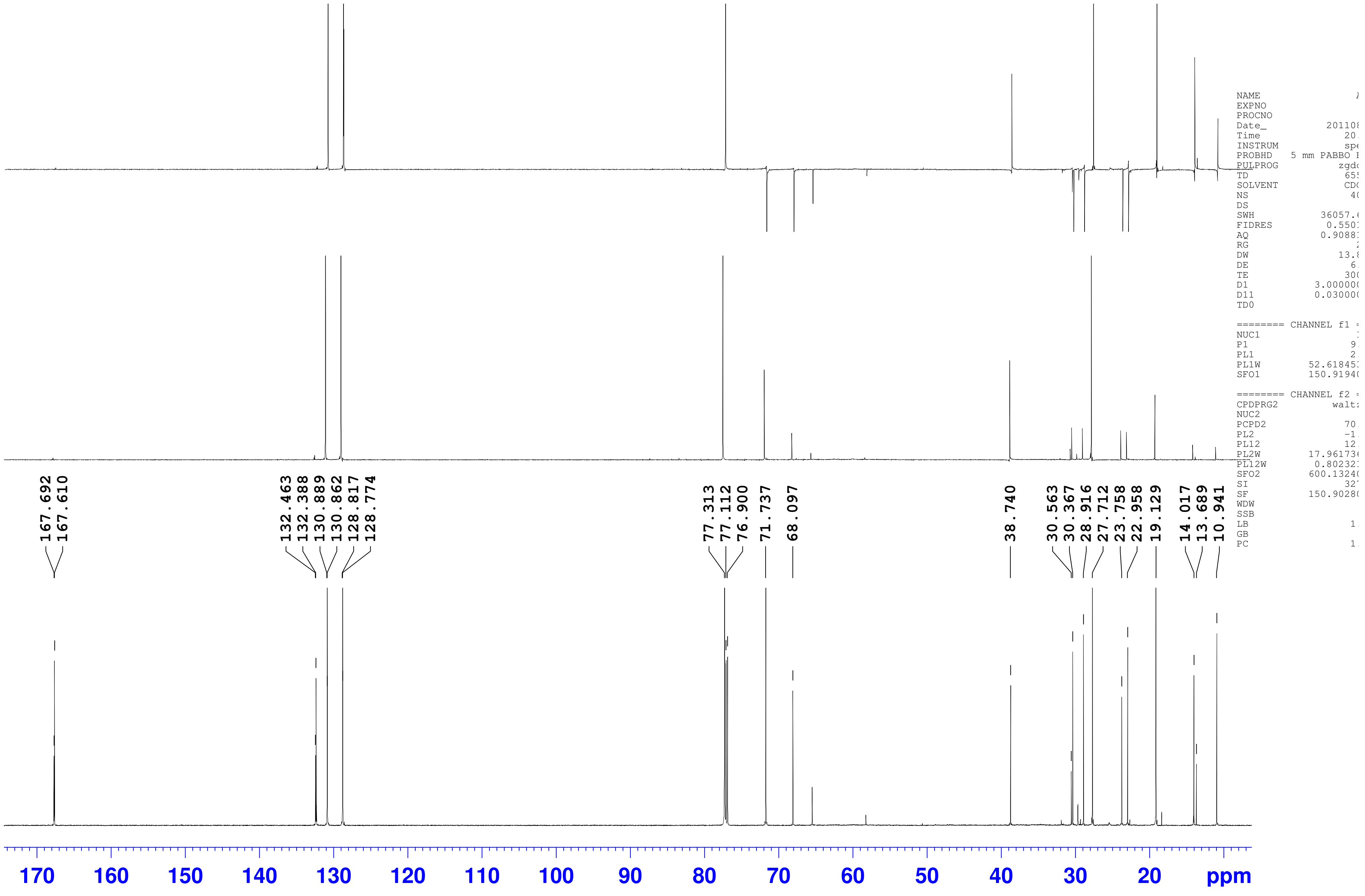

167.692
167.610
132.463
132.388
130.889
130.862
128.817
128.774
77.313
77.112
76.900
71.737
68.097
38.740
30.563
30.367
28.916
27.712
23.758
22.958
19.129
14.017
13.689
10.941
170 160 150 140 130 120 110 100 90 80 70 60 50 40 30 20 ppm
NAME
EXPNO
PROCNO
Date_
Time
INSTRUM
PROBHD 5 mm PABBO
PULPROG
TD
SOLVENT
NS
DS
SWH 36057.
FIDRES 0.550
AQ 0.9088
RG
DW 13.
DE
TE
D1 3.00000
D11 0.03000
TD0
======== CHANNEL f1
NUC1
P1
PL1
PL1W 52.61845
SFO1 150.9194
======== CHANNEL f2
CPDPRG2
NUC2
PCPD2
PL2
PL12
PL2W 17.96173
PL12W 0.80232
SFO2 600.1324
SI
SF 150.9028
WDW
SSB
LB
GB
PC
