# Supplementary material for: Discovery of an algicidal compound from Brevibacterium sp. BS01 and its effect on a harmful algal bloom-causing species, Alexandrium tamarense
Source: Front Microbiol. 2015 Nov 5;6:1235. doi: 10.3389/fmicb.2015.01235 (PMC4633486; doi:10.3389/fmicb.2015.01235)
Supplement: Supplementary Figure 8 — Distortionless Enhancement by Polarization Transfer (DEPT) spectrum of fraction C2 in CDCl3 (chemical shift: 11–174 ppm). [file Image8.PDF]

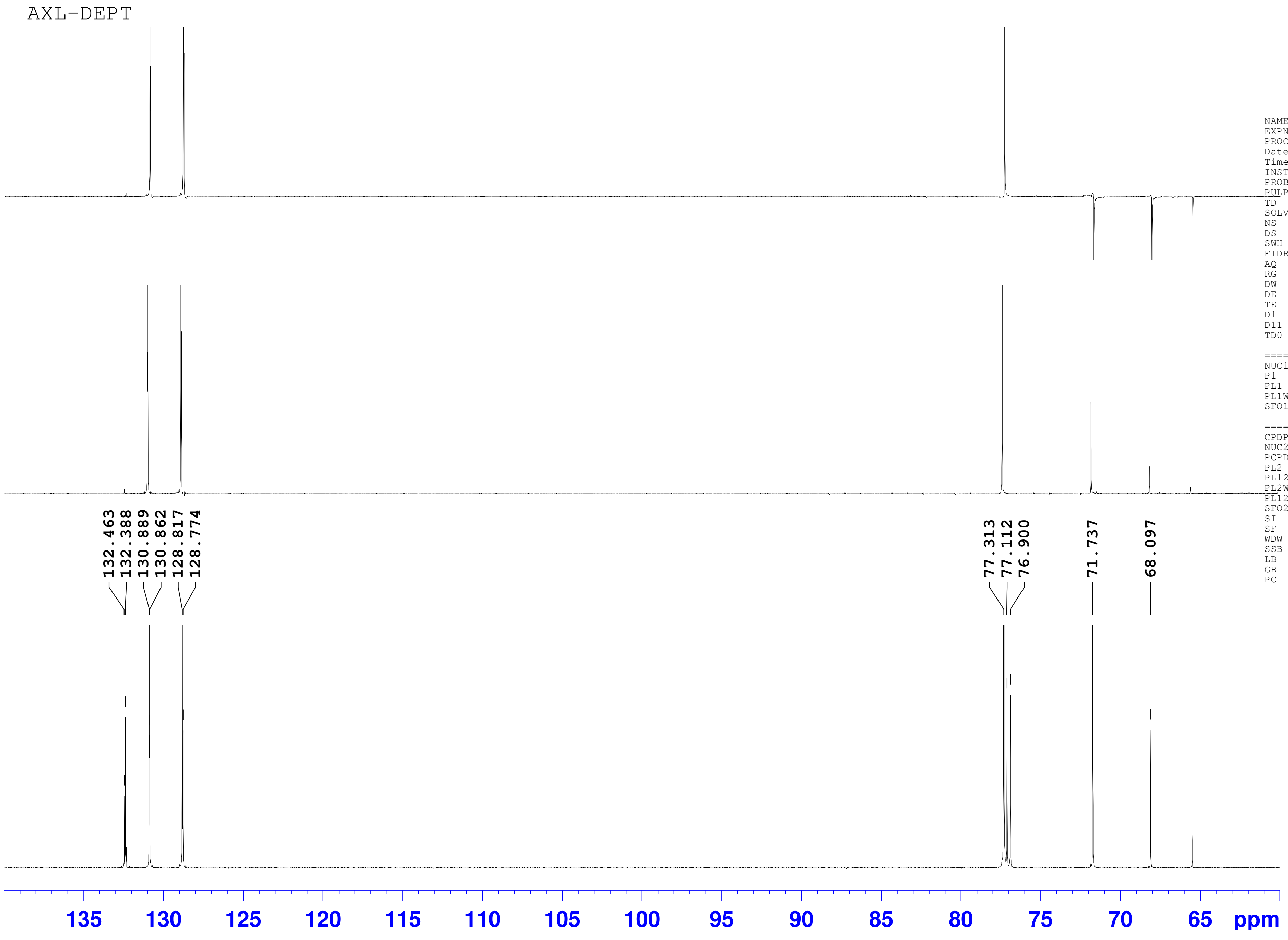

NAME	[illegible]
EXPNO	
PROCNO	
Date_	20110[illegible]
Time	20[illegible]
INSTRUM	sp[illegible]
PROBHD	5 mm PABBO [illegible]
PULPROG	zgd[illegible]
TD	65[illegible]
SOLVENT	CD[illegible]
NS	4[illegible]
DS	
SWH	36057.[illegible]
FIDRES	0.550[illegible]
AQ	0.9088[illegible]
RG	[illegible]
DW	13.[illegible]
DE	6[illegible]
TE	30[illegible]
D1	3.00000[illegible]
D11	0.03000[illegible]
TD0	
======== CHANNEL f1 [illegible]	
NUC1	[illegible]
P1	9[illegible]
PL1	2[illegible]
PL1W	52.61845[illegible]
SFO1	150.9194[illegible]
======== CHANNEL f2 [illegible]	
CPDPRG2	walt[illegible]
NUC2	
PCPD2	70[illegible]
PL2	-1[illegible]
PL12	12[illegible]
PL2W	17.96173[illegible]
PL12W	0.80232[illegible]
SFO2	600.1324[illegible]
SI	32[illegible]
SF	150.9028[illegible]
WDW	
SSB	
LB	1[illegible]
GB	
PC	1[illegible]
