# Supplementary material for: Discovery of an algicidal compound from Brevibacterium sp. BS01 and its effect on a harmful algal bloom-causing species, Alexandrium tamarense
Source: Front Microbiol. 2015 Nov 5;6:1235. doi: 10.3389/fmicb.2015.01235 (PMC4633486; doi:10.3389/fmicb.2015.01235)
Supplement: Supplementary Figure 9 — Distortionless Enhancement by Polarization Transfer (DEPT) spectrum of fraction C2 in CDCl3 (chemical shift: 60–140 ppm). [file Image9.PDF]

AXL-DEPT

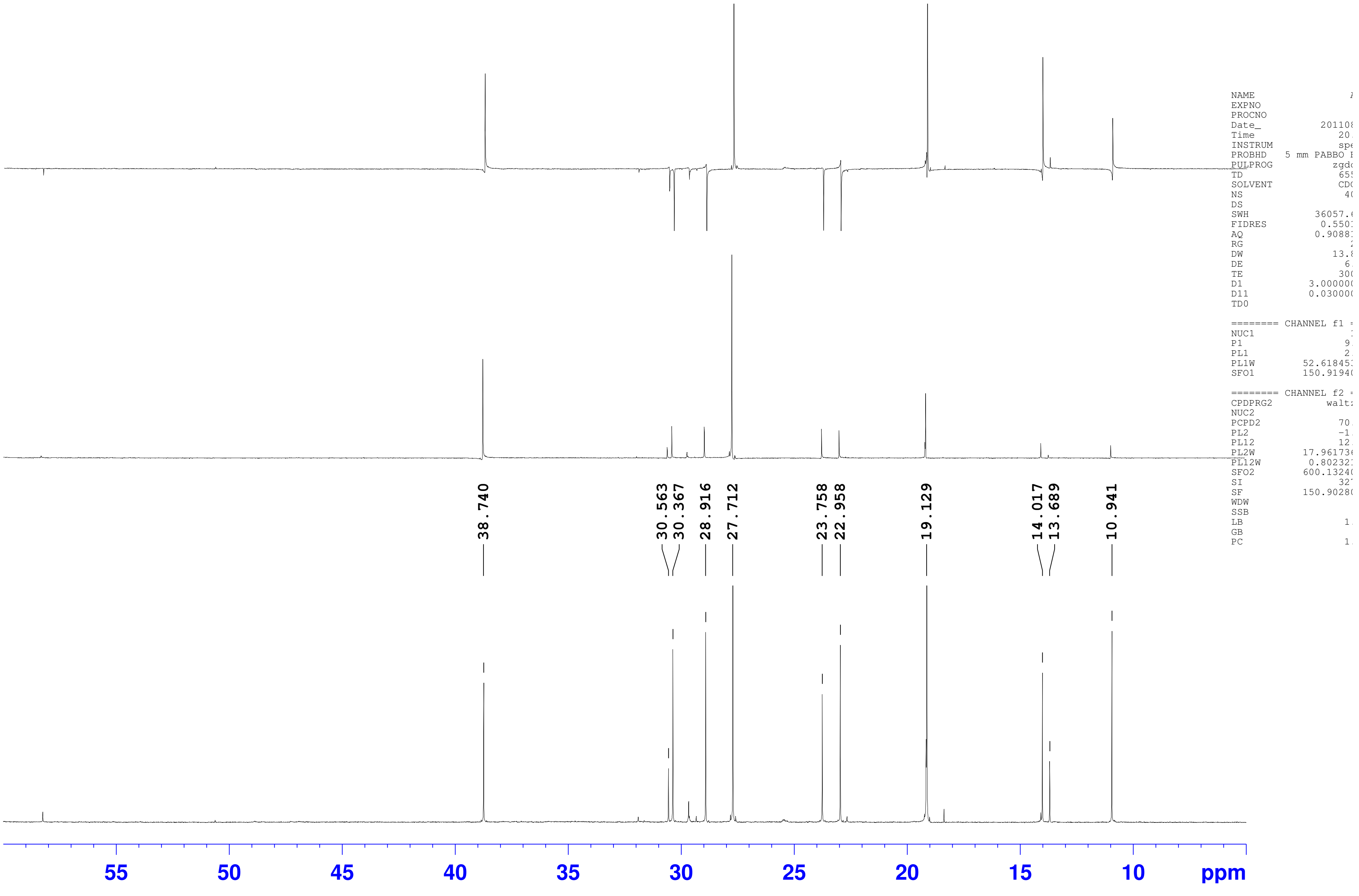
NAME
EXPNO
PROCNO
Date_ 20110
Time 20
INSTRUM sp
PROBHD 5 mm PABBO
PULPROG zgd
TD 65
SOLVENT CD
NS 4
DS
SWH 36057.
FIDRES 0.550
AQ 0.9088
RG
DW 13.
DE 6
TE 30
D1 3.00000
D11 0.03000
TD0
======== CHANNEL f1
NUC1
P1 9
PL1 2
PL1W 52.61845
SFO1 150.9194
======== CHANNEL f2
CPDPRG2 walt
NUC2
PCPD2 70
PL2 -1
PL12 12
PL2W 17.96173
PL12W 0.80232
SFO2 600.1324
SI 32
SF 150.9028
WDW
SSB
LB 1
GB
PC 1
38.740
30.563
30.367
28.916
27.712
23.758
22.958
19.129
14.017
13.689
10.941
55
50
45
40
35
30
25
20
15
10
ppm
