# Supplementary material for: Discovery of an algicidal compound from Brevibacterium sp. BS01 and its effect on a harmful algal bloom-causing species, Alexandrium tamarense
Source: Front Microbiol. 2015 Nov 5;6:1235. doi: 10.3389/fmicb.2015.01235 (PMC4633486; doi:10.3389/fmicb.2015.01235)
Supplement: Supplementary Figure 15 — HPLC analysis of the authentic standard ((2-isobutoxyphenyl)amine, 5 ppm) and the algicidal culture of BS01. The algicidal culture was prepared by extracting 200 mL culture for three times with equal volumes of ethyl acetate. [file Image15.PDF]

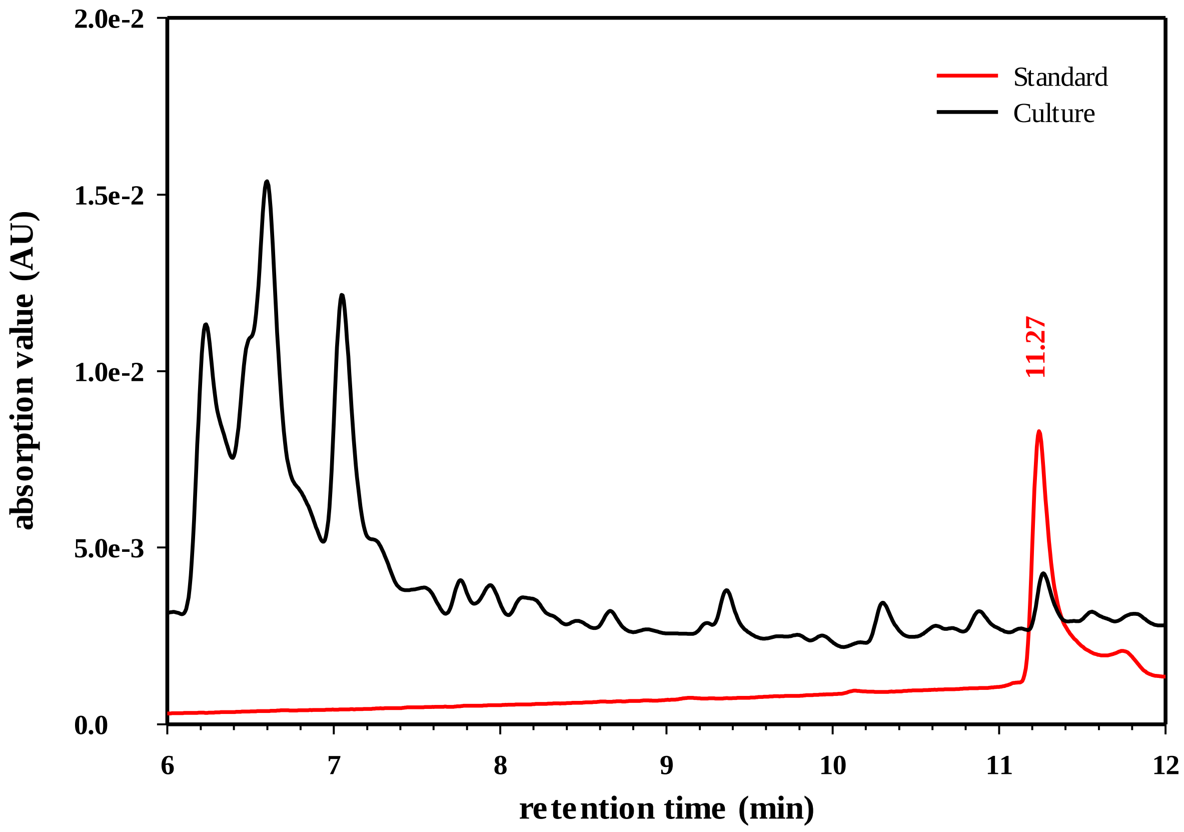

Fig. 15 HPLC analysis of the authentic standard ((2-isobutoxyphenyl)amine, 5 ppm) and the algicidal culture of BS01. The algicidal culture was prepared by extracting 200 mL culture for three times with equal volumes of ethyl acetate.
